# Profiling the TRPV4 ankyrin repeat domain interactome and its disruption by neuromuscular disease-causing mutations

**DOI:** 10.1016/j.jbc.2025.110991

**Published:** 2025-12-01

**Authors:** Alexis K. Loder, Gage P. Kosmanopoulos, William H. Aisenberg, Eric Cox, Alexis R. Meeker, Seth Blackshaw, Rachelle Gaudet, Ute A. Hellmich, Brett A. McCray, Charlotte J. Sumner, Jeremy M. Sullivan

**Affiliations:** 1 Department of Neurology, Johns Hopkins University School of Medicine, Baltimore, Maryland, USA; 2 Department of Neurology, University of Michigan Medical School, Ann Arbor, Michigan, USA; 3 Solomon H. Snyder Department of Neuroscience, Johns Hopkins University School of Medicine, Baltimore, Maryland, USA; 4 Department of Ophthalmology, Johns Hopkins University School of Medicine, Baltimore, Maryland, USA; 5 Department of Neurology, Institute for Cell Engineering, Kavli Neuroscience Discovery Institute, Johns Hopkins University, Baltimore, Maryland, USA; 6 Department of Molecular and Cellular Biology, Harvard University, Cambridge, Massachusetts, USA; 7 Institute of Organic Chemistry & Macromolecular Chemistry (IOMC), Friedrich Schiller University, Jena, Germany; 8 Cluster of Excellence "Balance of the Microverse", Friedrich Schiller University Jena, Jena, Germany; 9 Department of Genetic Medicine, Johns Hopkins University School of Medicine, Baltimore, Maryland, USA

**Keywords:** TRP channels, neurodegenerative disease, ubiquitination, GEF, calcium imaging

## Abstract

The ankyrin repeat is one of the most abundant protein-protein interaction motifs in eukaryotes yet occurs in only a small number of ion channels. These channels are all members of the transient receptor potential (TRP) superfamily and contain prominent ankyrin repeat domains (ARDs) in their cytoplasmic N termini. In transient receptor potential vanilloid 4 (TRPV4), the importance of this domain has been highlighted by the finding that gain-of-function neuromuscular disease-causing missense mutations cluster on the ARD surface. Little is known currently about the extent of the TRPV4-ARD interactome, nor how it may be altered by disease-causing mutations. Here, we utilized a human proteome microarray to profile the ARD interactomes of WT and mutant TRPV4. Probing of the microarray with TRPV4^WT^-ARD revealed 78 interactors, including proteins related to ubiquitination and small GTPase signaling, such as the ubiquitin ligase NEDD4L and the RhoGEF ARHGEF10. In parallel experiments, we also identified the deubiquitinase OTUB2 as an interactor of the proximal N terminus. Comparison of the ARD interactomes of WT and mutant TRPV4 revealed 21 interactions affected by disease-causing mutations. Strikingly, one of these interactors, ARHGEF10, is also mutated in neuromuscular disease. Cell-based studies confirmed that ARHGEF10 exhibits a reduced capacity to coimmunoprecipitate with mutant TRPV4. Furthermore, calcium imaging studies demonstrated that ARHGEF10 overexpression suppressed TRPV4^WT^ channel activity, but that this inhibition is abrogated by disease-causing mutations. Together, these findings provide insights into the functional roles of an ion channel ARD, as well as their disruption in disease, and offer a resource for future cell-based studies.

The ankyrin repeat sequence motif is a protein-protein interaction platform found in myriad proteins with diverse functions. Ankyrin repeat domains (ARDs) consist of tandem ankyrin repeats of varying length comprising a conserved hydrophobic core surrounded by surface residues that are individual to each protein. These protein-specific surface residues enable ARDs to mediate interactions with high specificity (1, 2, 3, 4). Despite being one of the most abundant protein-protein interaction motifs in eukaryotic proteins, ARDs occur in only a select number of membrane channels. All these channels are members of the transient receptor potential (TRP) superfamily and contain prominent ARDs in their cytoplasmic N termini (2). ARDs are stable in isolation and were among the first domains of TRP channels to be structurally characterized (5, 6, 7, 8, 9). The functional importance of these domains has been highlighted by the discovery of a cluster of neuromuscular disease-causing mutations on a cytosol-facing surface of the ARD in transient receptor potential vanilloid 4 (TRPV4) (5, 10, 11, 12).

TRPV4 is a non-selective calcium-permeable cation channel that functions primarily as a homotetramer at the plasma membrane, where it is activated by a variety of environmental and chemical stimuli (13). Dominant (or rarely recessive) gain-of-function missense mutations in TRPV4 cause three forms of hereditary neuromuscular disease: Charcot-Marie-Tooth disease type 2C, congenital distal spinal muscular atrophy, and scapuloperoneal spinal muscular atrophy (5, 10, 11, 14, 15, 16, 17, 18, 19). These disorders, although clinically heterogeneous, typically cause weakness of the proximal and distal aspects of the limbs, as well as diaphragm and vocal fold weakness (17, 20). No disease-modifying treatment is currently available.

Structurally, each TRPV4 subunit comprises six transmembrane segments with a pore loop between the fifth and sixth segments and intracellular N and C termini (Fig. 1*A*). The cytoplasmic N terminus represents approximately half of the TRPV4 protein and comprises an intrinsically disordered region (IDR) (21), six ankyrin repeats, and a linker to the transmembrane region (5, 12, 22, 23). Together, the six ankyrin repeats form a hand-shaped ARD with a concave “palm” surface and a convex surface analogous to the back of the hand (2, 9). Neuromuscular disease-causing mutations (*e*.*g*. R269C, R315W) occur primarily at exposed arginine residues clustered on the convex face of the ARD (5, 12) (Fig. 1*B*). The ability of TRPV4 to bind to known ARD interactors calmodulin and ATP is not altered by neuromuscular disease-causing mutations, suggesting that the mutations do not substantially alter the fold or stability of the ARD (5, 9). In addition to hereditary neuromuscular disease, distinct missense mutations of TRPV4 are associated with several forms of skeletal dysplasia (24). Whereas neuromuscular disease-causing mutations cluster in the TRPV4-ARD, mutations associated with skeletal dysplasia occur throughout the protein (24). Interestingly, those skeletal dysplasia-causing mutations located within the ARD (*e*.*g*. D333G) occur primarily on the concave (“palm”) face, the opposite face to the neuromuscular disease-causing mutations (9, 12) (Fig. 1*B*).

**Figure 1:**
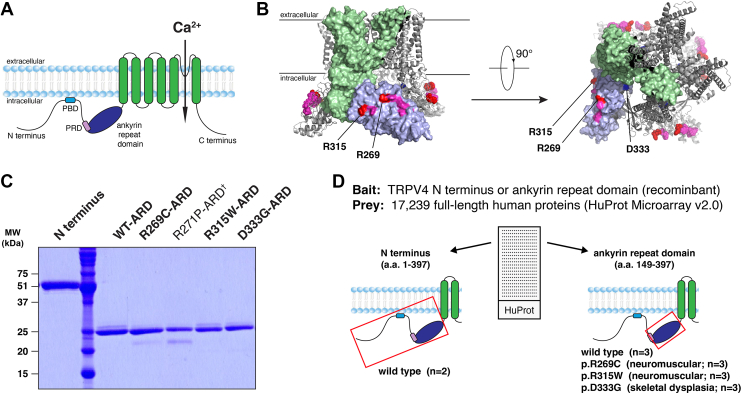
**Identification of candidate interactors of the TRPV4 WT and mutant ARD utilizing a human protein microarray**. *A*, schematic of a TRPV4 protomer with the key functional domains highlighted. The PIP_2_-binding domain (PBD) and proline-rich domain (PRD) reside within the N-terminal intrinsically disordered region. *B*, schematic of the TRPV4 homotetramer with the transmembrane regions (*green*) and ARD (*purple*) of a single subunit highlighted. *Red*, the R269 and R315 residues mutated in TRPV4-mediated neuromuscular disease examined in the present study; *pink*, additional residues mutated in TRPV4-mediated neuromuscular disease; *blue*, the D333 residue mutated in TRPV4-mediated skeletal dysplasia (*Homo sapiens*, PDB: 8FC9). *C*, Coomassie blue-stained polyacrylamide gel showing the purified TRPV4 N terminus and ARD constructs. The R271P-ARD construct (*dagger*) was not examined further in the present study. *D*, schematic summarizing the experimental design adopted to identify potential interactors of the TRPV4 N terminus and ARD utilizing the HuProt Microarray. ARD, ankyrin repeat domain; TRPV4, transient receptor potential vanilloid 4.

Little is known about how different mutations in TRPV4 result in such diverse disease phenotypes (24, 25). Heterologous expression studies suggest that both neuromuscular disease- and skeletal dysplasia-causing TRPV4 mutants exhibit normal expression levels but increased channel activity (24, 25, 26). Recent analyses of cryo-EM structures suggest that many skeletal dysplasia-causing mutations located outside the ARD may directly influence the TRPV4 pore gate, whereas those in the ARD may impact inter-subunit interactions (27). In contrast, neuromuscular disease-causing mutations occur on the outer surface of the TRPV4 tetramer, suggesting that the pathogenic consequences of these mutations may result from altered interactions with ARD binding partners (10, 12, 22, 27, 28).

In recent work, we identified the small GTPase RhoA as a robust interactor of the TRPV4-ARD (28), and this was subsequently confirmed with reports of the TRPV4-RhoA complex by cryo-EM (12, 22). RhoA is a key regulator of the actin cytoskeleton that cycles between active GTP-bound and inactive GDP-bound conformations, a process tightly regulated by guanine nucleotide exchange factors (GEFs), GTPase-activating proteins (GAPs), and guanine nucleotide dissociation inhibitors. TRPV4 interacts preferentially with RhoA in its inactive (GDP-bound) conformation, resulting in suppression of RhoA activation in a manner analogous to a guanine nucleotide dissociation inhibitors (28). Strikingly, neuromuscular disease- but not skeletal dysplasia-causing mutations disrupt TRPV4-RhoA binding and the capacity of TRPV4 to inhibit RhoA activation (12, 28). These findings suggest that regulation of small GTPase signaling may represent a key function of the TRPV4-ARD and that disruption of this function may play a role in the pathogenesis of TRPV4-mediated neuromuscular disease. Little is known currently, however, about the extent of the TRPV4-ARD interactome, nor how it may be altered by neuromuscular disease-causing mutations.

The goals of the present study were twofold: (1) to characterize the interactome of the WT human TRPV4-ARD, and (2) to assess the effects of neuromuscular disease-causing mutations on these interactions. To limit influence from cell type-specific expression patterns of target proteins, we utilized a human protein microarray to profile and contrast the interactome of recombinant TRPV4^WT^-ARD with those of neuromuscular disease- (TRPV4^R269C^-ARD, TRPV4^R315W^-ARD) and skeletal dysplasia- (TRPV4^D333G^-ARD) causing mutants. The findings from these cell-free experiments were supported by cell-based studies of select identified microarray interactors, including coimmunoprecipitation experiments, ubiquitination assays, and ratiometric calcium imaging.

## Results

### Characterizing the TRPV4 interactome by protein microarray screening

**Figure 2:**
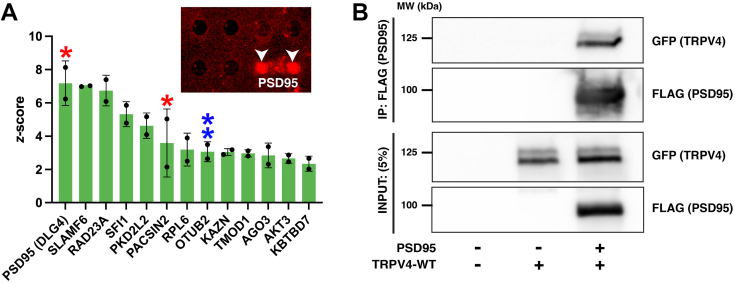
**Protein interactome of the TRPV4^WT^ N terminus**. *A*, candidate interactors of the TRPV4^WT^ N terminus. Two previously identified TRPV4 interactors (PSD95 and PACSIN2) are identified by *red asterisks*. The *blue double asterisks* highlight the deubiquitinase OTUB2. The inset shows the duplicate protein spots for PSD95 (*white arrowheads*) following immunodetection of the V5 tag (*red*) present on the TRPV4^WT^ N-terminus peptide. Plot shows mean ± SD. *B*, immunoblots demonstrating coimmunoprecipitation of TRPV4^WT^-GFP and PSD95-FLAG from transiently cotransfected HEK293T cells. TRPV4, transient receptor potential vanilloid 4.

To identify novel binding partners of the TRPV4^WT^-ARD in an unbiased manner, we utilized the HuProt proteome microarray to screen for interactions between this domain and >17,000 full-length human proteins (in duplicate; Fig. 1, *C* and *D*). To first evaluate the applicability of this approach, the microarray was probed with recombinant TRPV4^WT^ N-terminal domain (IDR and ARD, amino acids 1–397), which contains a proline-rich domain (PRD, amino acids 121–134; Fig. 1*A*) that mediates well-characterized interactions with the SH3 domains of the three PACSIN isoforms (PACSIN1-3) (5, 29, 30, 31). Of the three isoforms, both PACSIN1 and PACSIN2 are represented in the HuProt microarray. Microarray slides were probed with purified, V5-tagged TRPV4^WT^ N-terminal domain (Fig. 1, *C* and *D*), which was then detected using an Alexa fluorophore-conjugated V5 antibody. Labelled microarrays were imaged and analyzed to calculate a *z*-score (*z*) for each protein spot based on its background-corrected signal intensity. Candidate binding partners were identified as proteins for which the duplicate spots had background-corrected signal intensities ≥2 SDs above the mean value for the slide (*i*.*e*.*, z* ≥ 2). Analysis of both microarray slides probed with the TRPV4^WT^ N-terminal domain identified PACSIN2 as a positive binding partner (Fig. 2*A*), providing proof-of-concept evidence supporting the utility of this screening approach.

In addition to PACSIN2, analysis of the two microarray slides identified an additional 12 candidate binding partners of the TRPV4 N terminus. The most prominent of these hits was PDS95 (encoded by the *DLG4* gene; Fig. 2*A*), a neuronal scaffolding protein identified recently as a TRPV4 interactor (32). TRPV4 and PSD95 have also been shown to colocalize in cultured primary mouse hippocampal neurons (33). To independently assess whether PSD95 associates with TRPV4, we performed coimmunoprecipitation (co-IP) assays in HEK293T cells transiently cotransfected with epitope-tagged forms of both proteins (TRPV4-GFP and PSD95-FLAG). Consistent with published work (32), we validated that TRPV4-GFP coimmunoprecipitated with PSD95-FLAG (Fig. 2*B*).

### Identification of protein interactors of the TRPV4^WT^-ARD

**Figure 3:**
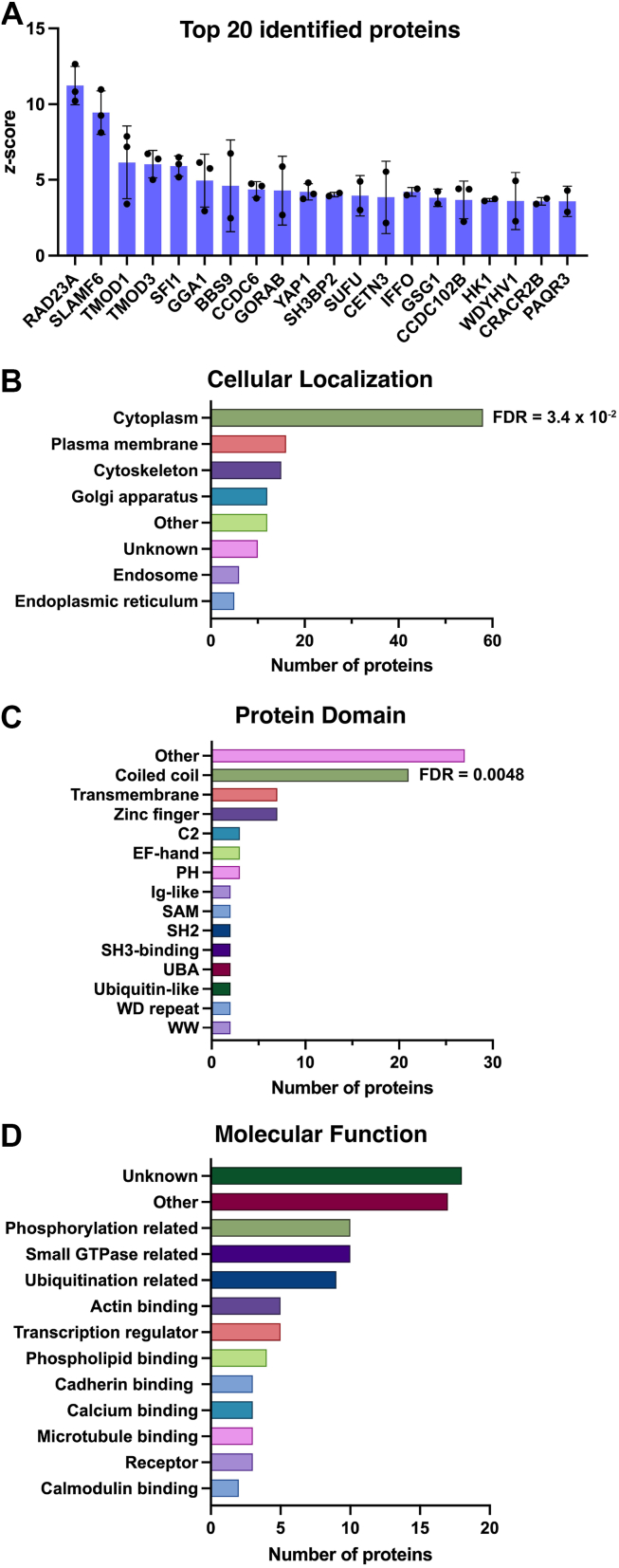
**Protein interactome of the TRPV4^WT^-ARD**. *A*, top 20 candidate interactors of the TRPV4^WT^-ARD based on mean *z*-score. The remaining interactors are listed in Table 1. Plot shows mean ± SD. *B*–*D*, Summary of the cellular localization (*B*), protein domains (*C*), and molecular function (*D*) of the candidate interactors of the TRPV4^WT^-ARD based on UniProt and Gene Ontology (GO) annotations and the supporting literature. FDR values indicating significant enrichment for GO categories utilizing the PANTHER database (*B*, GO:0005829, cytosol) or UniProt keywords utilizing the STRING database (*C*, KW-0175, coiled coil domain) are also shown. ARD, ankyrin repeat domain; GO, Gene Ontology; TRPV4, transient receptor potential vanilloid 4.

To identify candidate binding partners of the TRPV4^WT^-ARD, protein microarray slides (n = 3) were probed with V5-tagged WT-ARD. Candidates were identified as proteins on two of three microarray slides with duplicate spots showing background-corrected signal intensities with a *z*-score ≥ 2. Utilizing these criteria, 107 candidate binding proteins were identified. Due to the well-established localization of TRPV4 to the plasma membrane (13), candidate interactors that localize solely to nuclear, inner mitochondrial, or extracellular compartments were excluded from further analysis (n = 26 proteins; see Table S1). The remaining 78 candidate binding partners are detailed in Table 1, with the 20 proteins exhibiting the highest signal intensities also shown in Figure 3*A*. Six of the candidate binding partners of the full TRPV4^WT^ N-terminal domain (PSD95, PKD2L2, PACSIN2, OTUB2, AGO3, AKT3) were not identified on the microarray when probed with the TRPV4^WT^-ARD alone, suggesting that they bind specifically to the IDR.

**Table 1:** Candidate binding partners of the TRPV4^WT^-ARD

Protein (UniProtKB)	Gene	Mean *z*-score	SD	# of chips (n = 3 total)
UV excision repair protein RAD23 homolog A (P54725)	*RAD23A*	11.23	1.26	3
SLAM family member 6 (Q96DU3)	*SLAMF6*	9.45	1.43	3
Tropomodulin-1 (P28289)	*TMOD1*	6.16	2.41	3
Tropomodulin-3 (Q9NYL9)	*TMOD3*	6.04	0.91	3
Protein SFI1 homolog (A8K8P3)	*SFI1*	5.91	0.68	3
ADP-ribosylation factor-binding protein GGA1 (Q9UJY5)	*GGA1*	4.96	1.75	3
Protein PTHB1 (Q3SYG4)	*BBS9*	4.61	3.03	2
Coiled-coil domain-containing protein 6 (Q16204)	*CCDC6*	4.37	0.52	3
RAB6-interacting golgin (Q5T7V8)	*GORAB*	4.29	2.27	2
Transcriptional coactivator YAP1 (P46937)	*YAP1*	4.21	0.54	3
SH3 domain-binding protein 2 (P78314)	*SH3BP2*	4.03	0.14	2
Suppressor of fused homolog (Q9UMX1)	*SUFU*	3.95	1.33	2
Centrin-3 (O15182)	*CETN3*	3.85	2.39	2
Intermediate filament family orphan 1 (Q0D2I5)	*IFFO1*	3.83	0.67	3
Germ cell-specific gene 1 protein (Q2KHT4)	*GSG1*	3.82	0.58	2
Coiled-coil domain-containing protein 102B (Q68D86)	*CCDC102B*	3.67	1.24	3
Hexokinase-1 (P19367)	*HK1*	3.67	0.10	2
Protein N-terminal glutamine amidohydrolase (Q96HA8)	*WDYHV1*	3.61	1.88	2
EF-hand calcium-binding domain-containing protein 4A (Q8N4Y2)	*CRACR2B*	3.59	0.25	2
Progestin and adipoQ receptor family member 3 (Q6TCH7)	*PAQR3*	3.58	1.00	2
Ermin (Q8TAM6)	*ERMN*	3.57	0.88	3
Coiled-coil domain-containing protein 181 (Q5TID7)	*CCDC181*	3.51	0.14	2
Ras-related protein Rab-7L1 (O14966)	*RAB29*	3.47	0.90	3
Rab-3A-interacting protein (Q96QF0)	*RAB3IP*	3.44	0.65	3
Alpha-adducin (P35611)	*ADD1*	3.42	0.84	2
RILP-like protein 1 (Q5EBL4)	*RILPL1*	3.40	0.55	3
OTU domain-containing protein 5 (Q96G74)	*OTUD5*	3.30	0.08	2
Carbonic anhydrase 13 (Q8N1Q1)	*CA13*	3.25	0.97	2
Telomeric repeat-binding factor 2-interacting protein 1 (Q9NYB0)	*TERF2IP*	3.21	0.51	2
2-acylglycerol O-acyltransferase 2 (Q3SYC2)	*MOGAT2*	3.12	0.77	2
Dystrobrevin alpha (Q9Y4J8)	*DTNA*	3.12	1.08	3
Histone-lysine N-methyltransferase SMYD3 (Q9H7B4)	*SMYD3*	3.09	0.23	2
Translin-associated factor X-interacting protein 1 (Q2TAA8)	*TSNAXIP1*	3.05	0.04	2
Kazrin (Q674X7)	*KAZN*	3.04	0.55	3
Rho guanine nucleotide exchange factor 10 (O15013)	*ARHGEF10*	3.01	0.19	2
ADP-ribosylation factor GTPase-activating protein 1 (Q8N6T3)	*ARFGAP1*	2.99	0.57	2
60S ribosomal protein L6 (Q02878)	*RPL6*	2.99	0.70	3
GATOR complex protein WDR24 (Q96S15)	*WDR24*	2.97	0.05	2
DNA fragmentation factor subunit alpha (O00273)	*DFFA*	2.96	0.92	2
Ciliary microtubule inner protein 4 (O43247)	*CIMIP4*	2.95	1.02	3
Short coiled-coil protein (Q9UIL1)	*SCOC*	2.92	0.84	2
Dual adapter for phosphotyrosine and 3-phosphotyrosine and 3-phosphoinositide (Q9UN19)	*DAPP1*	2.92	0.87	2
Uncharacterized protein C6orf141 (Q5SZD1)	*C6orf141*	2.91	0.47	3
Microtubule-associated protein 9 (Q49MG5)	*MAP9*	2.90	0.19	2
Armadillo-like helical domain containing protein 1 (Q6PIY5)	*ARMH1*	2.88	0.09	2
Eukaryotic translation initiation factor 3 subunit J (O75822)	*EIF3J*	2.85	0.38	2
Coiled-coil domain-containing protein 28A (Q8IWP9)	*CCDC28A*	2.81	0.76	2
Myotubularin-related protein 2 (Q13614)	*MTMR2*	2.80	1.09	2
Sphingolipid delta(4)-desaturase/C4-monooxygenase DES2 (Q6QHC5)	*DEGS2*	2.79	0.22	2
3-hydroxybutyrate dehydrogenase type 2 (Q9BUT1)	*BDH2*	2.79	0.34	2
Synaptotagmin-like protein 2 (Q9HCH5)	*SYTL2*	2.76	0.31	2
E3 ubiquitin-protein ligase NEDD4-like (Q96PU5)	*NEDD4L*	2.73	0.75	2
ADP-ribosylation factor-like protein 10 (Q8N8L6)	*ARL10*	2.71	0.65	2
Huntingtin-interacting protein K (Q9NX55)	*HYPK*	2.71	0.49	2
Arf-GAP with Rho-GAP domain, ANK repeat and PH domain-containing protein 1 (Q96P48)	*ARAP1*	2.70	0.62	2
SH3 domain-containing protein 19 (Q5HYK7)	*SH3D19*	2.69	0.87	2
LOC554174 protein (Q96Gl5)	LOC554174	2.66	0.07	2
Deoxycytidylate deaminase (P32321)	*DCTD*	2.64	0.33	2
Kelch repeat and BTB domain-containing protein 7 (Q8WVZ9)	*KBTBD7*	2.63	0.40	2
LIM domain-containing protein 1 (Q9UGP4)	*LIMD1*	2.60	0.61	3
14-3-3 protein eta (Q04917)	*YWHAH*	2.60	0.35	2
Protein FAM104B (Q5XKR9)	*FAM104B*	2.60	0.32	2
LBH domain-containing protein 1 (Q9BQE6)	*LBHD1*	2.59	0.53	2
NADH-cytochrome b5 reductase 2 (Q6BCY4)	*CYB5R2*	2.23	0.47	2
Cysteine-rich DPF motif domain-containing protein 1 (Q6NVV7)	*CDPF1*	2.50	0.39	2
Myosin light chain 1/3, skeletal muscle isoform (P05976)	*MYL1*	2.46	0.15	2
Eyes absent homolog 4 (O95677)	*EYA4*	2.44	0.10	2
Kin of IRRE-like protein 3 (Q8IZU9)	*KIRREL3*	2.44	0.30	2
UV excision repair protein RAD23 homolog B (P54727)	*RAD23B*	2.39	0.10	2
Uncharacterized protein C4orf36 (Q96KX1)	*C4orf36*	2.39	0.05	2
Small proline-rich protein 2B (P35325)	*SPRR2B*	2.37	0.14	2
E3 ubiquitin-protein ligase COP1 (Q8NHY2)	*COP1*	2.37	0.13	2
NIF3-like protein 1 (Q9GZT8)	*NIF3L1*	2.35	0.04	2
LIM and cysteine-rich domains protein 1 (Q9NZU5)	*LMCD1*	2.30	0.13	3
Polyadenylate-binding protein-interacting protein 2 (Q9BPZ3)	*PAIP2*	2.22	0.01	2
Rab11 family-interacting protein 1 (Q6WKZ4)	*RAB11FIP1*	2.18	0.16	3
Vacuolar protein sorting-associated protein 53 homolog (Q5VIR6)	*VPS53*	2.15	0.16	2
Receptor-type tyrosine-protein phosphatase O (Q16827)	*PTPRO*	2.15	0.01	2

SD, standard deviation.

Analysis of the UniProt and Gene Ontology (GO) annotations and supporting literature for the 78 candidate binding partners of the TRPV4^WT^-ARD revealed that these proteins localize primarily to the cytoplasm (74%, n = 58), plasma membrane (19%, n = 15), cytoskeleton (19%, n = 15), and secretory pathway (endoplasmic reticulum & Golgi apparatus; 22%, n = 17; Fig. 3*B*, Table S2). As several of these proteins localize to multiple subcellular compartments (Table S2), the sum of the percentages listed above is greater than 100%. Of the 15 proteins exhibiting plasma membrane localization, one is an E3 ubiquitin ligase (NEDD4L), three are transmembrane proteins (KIRREL3, PTPRO, SLAMF6), six are adaptor/scaffold proteins (ADD1, DAPP1, DTNA, LIMD1, SH3D19, YWHAH), three are effector proteins (ARAP1, SYTL2, YAP1), and two associate with primary cilia (BBS9, RILPL1). Analysis of GO categories utilizing the PANTHER database identified significant enrichment of the 78 candidate binding proteins within the cytoplasm (GO:0005829; FDR = 3.4 x 10^-2^; Fig. 3*B*), consistent with the exclusion of candidate interactors that localize solely to nuclear, inner mitochondrial, or extracellular compartments. Significant enrichment, however, was not observed within other subcellular compartments.

An analysis of protein-protein interactions between individual interactors, performed using the STRING database (interaction score >0.9), identified seven interactions (Fig. S1): ADD1-TMOD3, ADD1-TMOD1, CETN3-SFI1, RAB3IP-BBS9, RAD23A-RAD23B, YWHAH-NEDD4L, and YWHAH-YAP1. These findings provide initial insights into putative protein complexes in which TRPV4 may form part. Structurally, the proteins comprising the TRPV4^WT^-ARD microarray interactome contain diverse protein domains (Fig. 3*C*), including regions important for lipid binding (*e*.*g*., C2 domains and Pleckstrin Homology domains), calcium binding (*e*.*g*., EF-hand domains) and protein-protein interactions (*e*.*g*., coiled coil domains, SAM domains, SH2 domains, and WW domains). The full list of domains present in each candidate binding protein is detailed in Table S3. Enrichment analysis performed utilizing the STRING database to assess UniProt “Family & Domain” Annotated Keywords identified significant enrichment only of coiled coil domains within the 78 candidate binding partners (UniProt Keyword-0175; FDR = 0.0048; Fig. 3*C*). The coiled coil is a common structural motif comprising two or more supercoiled α-helices that serves a variety of molecular functions (34, 35), including mediating protein-protein interactions (36, 37). Sequence analysis using the PRATT and ScanProSite software tools did not identity enrichment of conserved sequences within the group of 78 proteins.

The microarray interactome of the TRPV4^WT^-ARD also comprises proteins with diverse molecular functions (Fig. 3*D*; Table S4). Analysis of GO categories using the PANTHER database did not detect significant enrichment of proteins involved in specific biological molecular functions (GO-Molecular Function). However, we noted several proteins with functions related to small GTPase biology (13%, n = 10), consistent with our recent finding that TRPV4 regulates the activity levels of the small GTPase RhoA (28). These proteins include the Rho GEF ARHGEF10 (described in further detail below), a Rho GAP (ARAP1), three proteins involved in ADP-ribosylation factor GTPase function (ARFGAP1, ARL10, GGA1), and five proteins involved in Rab GTPase function (RAB11FIP1, RAB29, RAB3IP, RILPL1, SYTL2). Three of the microarray interactors (4%) were observed to be calcium-binding proteins (CETN3, CRACR2B, MYL1), and thus of potential relevance to the role of TRPV4 in calcium signaling. Each of these proteins binds calcium *via* an EF-hand domain. We also noted the identification of NEDD4L, a HECT family E3 ubiquitin ligase (38), as a putative TRPV4 interactor. NEDD4L is known to localize and function at the plasma membrane due its C2 lipid-binding domain (39, 40) (Table S3). Interestingly, we recently demonstrated that NEDD4, a paralogue of NEDD4L, interacts with and ubiquitinates TRPV4 (30). Furthermore, we identified multiubiquitination as a key post-translational modification modulating TRPV4 channel activity (30).

### Coexpression of novel TRPV4 interactors alters TRPV4 ubiquitination

**Figure 4:**
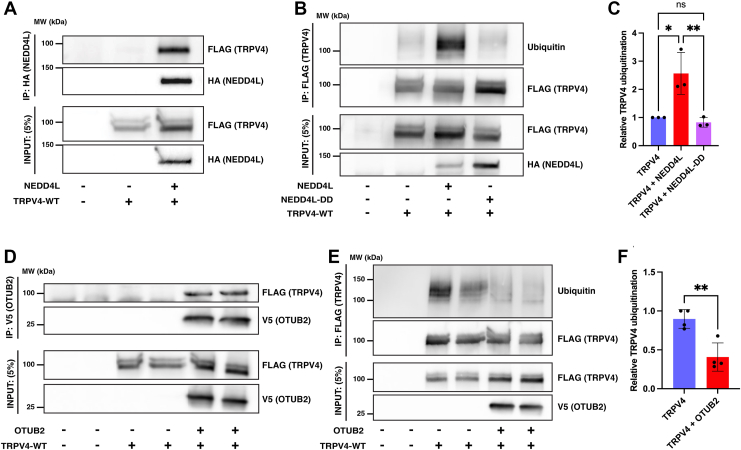
**Novel interactors alter TRPV4 ubiquitination levels**. *A*, coimmunoprecipitation of TRPV4-FLAG and HA-NEDD4L in transiently cotransfected HEK293T cells (n = 3 transfections/condition). *B*, TRPV4 is ubiquitinated in transfected HEK293T cells with overexpression of NEDD4L, but not with the catalytically inactive mutant NEDD4L-DD. *C*, densitometric quantification of *B* (n = 3 transfections/condition). Plot shows mean ± SD; one-way ANOVA, Tukey’s multiple comparison test; ns, not significant, ∗*p* = 0.012, ∗∗*p* = 0.007. *D*, coimmunoprecipitation of TRPV4-FLAG and OTUB2-V5 from transiently cotransfected HEK293T cells (n = 4 transfections/condition). *E*, TRPV4 is multiubiquitinated in cells without changing plasma membrane localization (30). Coexpression with OTUB2 results in deubiquitination. Shown are two independent samples per condition. *F*, densitometric quantification of *E* (n = 4 transfections/condition). Plot shows mean ± SD; unpaired two-sided *t* test, ∗∗*p* = 0.004. TRPV4, transient receptor potential vanilloid 4.

To further explore molecular pathways mediating TRPV4 ubiquitination, we investigated interactions between TRPV4^WT^ and NEDD4L by cotransfecting HEK293T cells with epitope-tagged forms of TRPV4^WT^ and NEDD4L. Co-IP assays revealed coimmunoprecipitation of TRPV4^WT^ (TRPV4^WT^-FLAG) and NEDD4L (HA-NEDD4L; Figure 4*A*). NEDD4L coexpression also resulted in a significant increase in TRPV4^WT^ ubiquitination levels, as revealed by immunoblot analyses of immunoprecipitated TRPV4 (Fig. 4, *B* and *C*). In contrast, overexpression of NEDD4L containing a point mutation (C962A) that renders the enzyme catalytically inactive (NEDD4L-DD (41)) did not alter TRPV4^WT^ ubiquitination levels (Fig. 4, *B* and *C*).

Analysis of the microarray interactome datasets also identified the deubiquitinating enzyme OTUB2, a candidate binding partner of the TRPV4^WT^ IDR (*blue double asterisks*, Fig. 2*A*), as an additional interactor that could potentially modify TRPV4 ubiquitination levels. OTUB2 is a deubiquitinase of the ovarian tumor protease family (42). Consistent with the interaction observed in the protein microarray, co-IP assays in transiently cotransfected HEK293T cells revealed coimmunoprecipitation of epitope-tagged TRPV4 (TRPV4^WT^-FLAG) and OTUB2 (OTUB2-V5; Fig. 4*D*). Furthermore, coexpression of OTUB2 resulted in a significant reduction in TRPV4^WT^ ubiquitination levels (Fig. 4, *E* and *F*), demonstrating a functional interaction between the two proteins. Together, these data identify novel interactors of the TRPV4^WT^ ARD (NEDD4L) and IDR (OTUB2) with opposing effects on TRPV4 ubiquitination status.

### Identification of protein interactions disrupted by neuromuscular disease-causing mutations

**Figure 5:**
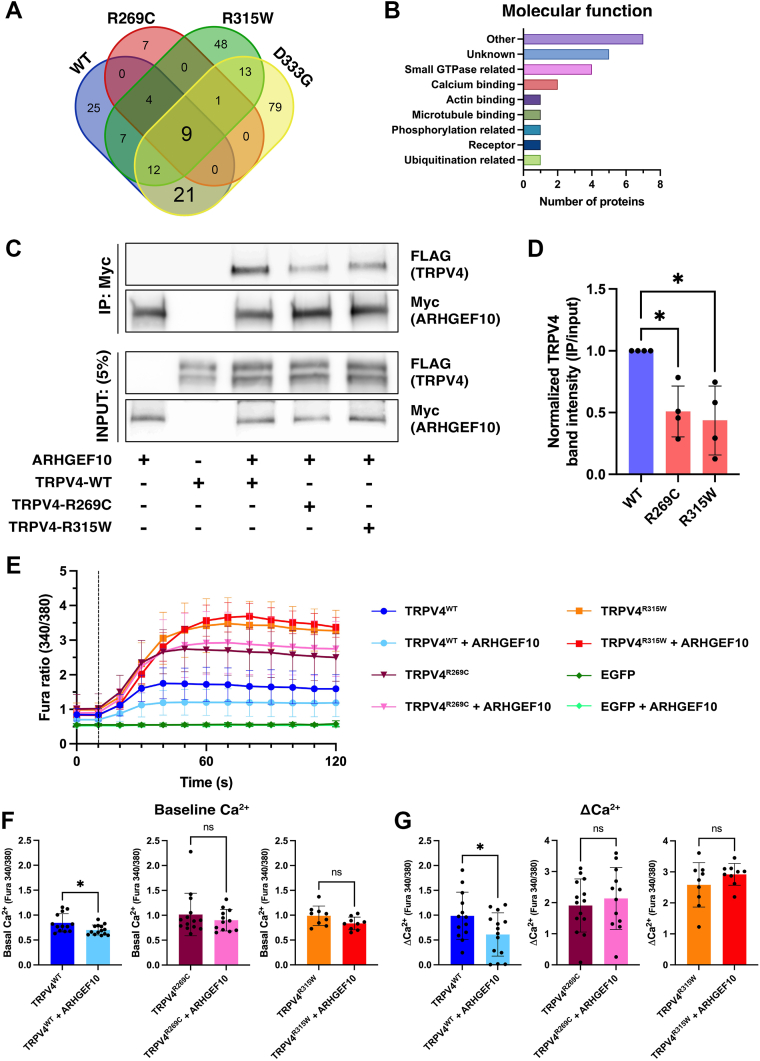
**Neuromuscular disease-causing mutations alter the TRPV4 ARD interactome**. *A*, Venn diagram of the candidate interactors of the TRPV4^WT^-ARD, TRPV4^R269C^-ARD, TRPV4^R315W^-ARD, and TRPV4^D333G^-ARD constructs. The 21 candidate interactors of the TRPV4^WT^-ARD and TRPV4^D333G^-ARD constructs that were not detected for the TRPV4^R269C^-ARD and TRPV4^R315W^-ARD constructs are listed in Table 2. *B*, molecular functions of the 21 candidate interactors absent from the microarrays probed with the TRPV4^R269C^-ARD and TRPV4^R315W^-ARD constructs based on UniProt and Gene Ontology (GO) annotations and the supporting literature. *C* and *D*, representative immunoblot images (*C*) and densitometry-based quantification (*D*) of coimmunoprecipitation experiments showing that neuromuscular disease-causing mutations in the TRPV4 ARD (R269C, R315W) reduced interactions with ARHGEF10. Plot shows mean ± SD; Kruskal-Wallis test, Dunn’s multiple comparison test, WT *versus* R269C, *p* = 0.043; WT *versus* R315W, *p* = 0.025. *E*, changes in the Fura-2 ratio (emission at 340/380 nm), an indicator of alterations in cytosolic free calcium, evoked in transfected MN-1 cells by application of hypotonic saline (*vertical dotted line*). N = 11 to 14 wells per condition, with >30 transfected cells per well. *F* and *G*, baseline cytosolic free calcium (*F*) and change in cytosolic free calcium (ΔCa^2+^) following hypotonic saline application (*G*) for the cells show in *E*. Plots show mean ± SD; *F*, unpaired two-sided *t* test with Welch’s correction, ns, not significant, ∗*p* = 0.022. *G*, unpaired two-sided *t* test; ns, not significant, ∗*p* = 0.043. ARD, ankyrin repeat domain; GO, Gene Ontology; TRPV4, transient receptor potential vanilloid 4.

To characterize alterations in the TRPV4^WT^-ARD interactome resulting from neuromuscular disease-causing mutations, human protein microarrays were probed with two mutant ARD constructs: TRPV4^R269C^-ARD and TRPV4^R315W^-ARD. These two mutants were selected for study as they both cause multiple forms of TRPV4-mediated neuromuscular disease (5, 11, 15, 16, 18, 43, 44, 45). As an additional control, we also examined the interactions of a TRPV4^D333G^-ARD construct containing a skeletal dysplasia-causing mutation on the concave face of the ARD (9, 46). The candidate binding partners identified for the three mutant ARD constructs are listed in Tables S5–7. Comparison of the candidate binding partners of the four ARD constructs (WT, R269C, R315W, D333G) revealed 21 proteins that were identified as putative interactors of both the WT-ARD and D333G-ARD but were absent from the interactomes of both neuromuscular disease mutant ARDs (Fig. 5*A*, Table 2): GORAB, CETN3, CRACR2B, PAQR3, ERMN, TERF2IP, MOGAT2, ARHGEF10, ARFGAP1, RPL6, DFFA, SCOC, EIF3J, BDH2, ARL10, HYPK, SH3D19, FAM104B, LBHD1, KIRREL3, and RAB11FIP1. No novel interactors specific to the two neuromuscular disease mutant ARDs were observed (Fig. 5*A*). The 21 proteins absent from both the R269C-ARD and R315W-ARD interactomes included proteins related to small GTPase biology (ARHGEF10, ARFGAP1, ARL10, RAB11FIP1) and calcium ion binding (CETN3, CRACR2B) (Fig. 5*B*). Analysis using the PANTHER database did not detect significant enrichment of proteins localizing to specific subcellular compartments or possessing specific molecular functions (GO-Cellular Component, GO-Molecular Function). Similarly, sequence analysis using the PRATT and ScanProSite software tools did not identity enrichment of conserved sequences.

**Table 2:** Candidate binding partners of TRPV4^WT^-ARD and TRPV4^D333G^-ARD absent from the microarrays probed with the TRPV4^R269C^-ARD and TRPV4^R315W^-ARD constructs

Protein (UniProtKB)	Gene	Mean *z*-score for WT-ARD
RAB6-interacting golgin (Q5T7V8)	*GORAB*	4.29
Centrin-3 (O15182)	*CETN3*	3.85
EF-hand calcium-binding domain-containing protein 4A (Q8N4Y2)	*CRACR2B*	3.59
Progestin and adipoQ receptor family member 3 (Q6TCH7)	*PAQR3*	3.58
Ermin (Q8TAM6)	*ERMN*	3.57
Telomeric repeat-binding factor 2-interacting protein 1 (Q9NYB0)	*TERF2IP*	3.22
2-acylglycerol O-acyltransferase 2 (Q3SYC2)	*MOGAT2*	3.12
Rho guanine nucleotide exchange factor 10 (O15013)	*ARHGEF10*	3.01
ADP-ribosylation factor GTPase-activating protein 1 (Q8N6T3)	*ARFGAP1*	2.99
60S ribosomal protein L6 (Q02878)	*RPL6*	2.99
DNA fragmentation factor subunit alpha (O00273)	*DFFA*	2.96
Short coiled-coil protein (Q9UIL1)	*SCOC*	2.92
Eukaryotic translation initiation factor 3 subunit J (O75822)	*EIF3J*	2.85
3-hydroxybutyrate dehydrogenase type 2 (Q9BUT1)	*BDH2*	2.79
ADP-ribosylation factor-like protein 10 (Q8N8L6)	*ARL10*	2.71
Huntingtin-interacting protein K (Q9NX55)	*HYPK*	2.71
SH3 domain-containing protein 19 (Q5HYK7)	*SH3D19*	2.69
Protein FAM104B (Q5XKR9)	*FAM104B*	2.60
LBH domain-containing protein 1 (Q9BQE6)	*LBHD1*	2.59
Kin of IRRE-like protein 3 (Q8IZU9)	*KIRREL3*	2.44
Rab11 family-interacting protein 1 (Q6WKZ4)	*RAB11FIP1*	2.18

### Neuromuscular disease-causing mutations disrupt functional interactions between TRPV4 and ARHGEF10

Strikingly, we noted that the Rho GEF ARHGEF10 (Rho guanine nucleotide exchange factor 10), one the 21 proteins exhibiting reduced interactions with R269C-ARD and R315W-ARD, has been linked to hereditary neuromuscular disease. Dominant mutations in ARHGEF10 are associated with forms of human neuromuscular disease (47, 48, 49, 50), as well as a form of neuromuscular disease in dogs that involves vocal fold paresis (51), a characteristic feature of TRPV4-mediated disease (11, 17, 20). To further explore a potential interaction between TRPV4 and ARHGEF10, we utilized MN-1 cells (a mouse motor neuron-neuroblastoma fusion cell line (52)) to perform a series of cell-based studies of TRPV4-ARHGEF10 interactions. Unlike HEK293T cells, MN-1 cells do not express endogenous TRPV4 and thus are well suited to transfection-based studies of mutant TRPV4 channels (12, 25, 26, 28). To further study interactions between TRPV4 and ARHGEF10, co-IP assays were performed in MN-1 cells transiently cotransfected with epitope-tagged forms of both proteins (TRPV4^WT^-FLAG and ARHGEF10-Myc). These studies demonstrated coimmunoprecipitation of TRPV4^WT^ and ARHGEF10 and confirmed reduced interaction of ARHGEF10 with the two neuromuscular disease-causing mutants (Fig. 5, *C* and *D*). To determine whether the TRPV4-ARHGEF10 interaction affected TRPV4 ion channel function, we performed ratiometric calcium imaging of MN-1 cells expressing TRPV4 alone or in combination with ARHGEF10. Notably, ARHGEF10 suppressed baseline and stimulated calcium levels in cells expressing TRPV4^WT^ (Fig. 5, *E*–*G*). However, the inhibitory effect of ARHGEF10 was absent in cells expressing the neuromuscular disease-causing mutants (Fig. 5, *E*–*G*). Together, these results suggest that reduced interaction of neuromuscular disease-causing TRPV4 mutants with ARHGEF10 and the resulting loss of ARHGEF10-mediated inhibition may contribute to the increased ion channel activity characteristic of these mutant channels.

## Discussion

ARDs are a characteristic feature of the cytosolic N termini of many TRP channels, including those of the TRPA, TRPC, and TRPV subfamilies (2). Roles for these domains in TRP channels have been defined in sensory transduction (53, 54) and in mediating protein-protein interactions (12, 28, 55, 56). The observation that neuromuscular disease-causing mutations in TRPV4 cluster on a cytosol-facing surface of the ARD has further highlighted the functional importance of these domains. Here, we have utilized a human protein microarray to gain insights into the protein interactome of the human TRPV4-ARD, as well as the TRPV4 N-terminal domain. Importantly, these studies identified known interactors of the TRPV4 N terminus, including the well-studied binding partner PACSIN2 (5, 29, 30, 31) and the recently identified interactor PSD95 (32), validating the utility of this approach. Probing of protein microarrays with human TRPV4-ARD identified 78 candidate binding partners of the WT ARD with diverse molecular functions, including small GTPase signaling and post-translational modifications. Comparison of the microarray interactomes of WT and mutant TRPV4-ARD identified 21 candidate binding partners exhibiting reduced interactions with neuromuscular disease-causing mutant forms of the ARD.

One difference between the microarray interactomes of the full N terminus and the TRPV4^WT^-ARD was the higher number of candidate binding partners identified for the ARD construct. In recent work utilizing crosslinking mass spectrometry, we have shown that the TRPV4 IDR and ARD exhibit extensive and dynamic interdomain interactions (21). The presence of the IDR in the full N terminus, therefore, may limit the amount of exposed ARD surface available for binding to other proteins. As the TRPV4 N terminus undergoes significant structural rearrangements during channel opening and closing (12, 57), interactions between the TRPV4-ARD and its binding partners may change dynamically within cells. Consistent with this concept, we recently demonstrated in cryo-EM studies that the relative occupancy of RhoA bound to the TRPV4^WT^-ARD changes progressively during channel gating (12). Although the co-IP experiments undertaken as part of this study were performed in the presence of a TRPV4-specific antagonist to limit effects of cytotoxicity due to TRPV4 overexpression, especially of the mutant channels (12, 28), an important subject for future studies will be to further explore effects of channel activity on the TRPV4-ARD interactome.

Although we and others have recently demonstrated that the small GTPase RhoA is a robust interactor of the TRPV4-ARD (12, 22, 28), it was not identified as an interactor in this study. The lack of TRPV4-RhoA binding observed in the microarray setting may be explained by our previous finding that TRPV4 binds preferentially to RhoA in its GDP-bound form (28). The Switch I and II loops of RhoA undergo conformational changes that are dependent upon GDP or GTP binding and which influence interactions with binding partners (58). One limitation of cell-free assays such as protein microarrays is that it is difficult in this format to replicate more complex, context-dependent forms of protein-protein interactions.

The cytoplasmic TRPV4 N terminus represents a key site for post-translational modifications regulating channel activity (59, 60, 61, 62). In recent work, we demonstrated that multiubiquitination of the TRPV4 N-terminal domain inhibits TRPV4 channel activity without altering cell surface expression levels (30). Further underscoring the pivotal role of the N-terminal domain in channel ubiquitination, here we identified the E3 ubiquitin ligase NEDD4L and the deubiquitinase OTUB2 as candidate interactors of the TRPV4^WT^-ARD and N-terminal IDR, respectively. Cell-based studies demonstrated co-IP of TRPV4 and NEDD4L in transiently cotransfected HEK293T cells, as well as a significant increase in channel ubiquitination following coexpression of the two proteins. This latter finding confirms a previous report demonstrating that NEDD4L overexpression can increase TRPV4 ubiquitination levels (63). Coexpression studies also validated interactions between TRPV4^WT^ and OTUB2, a broadly expressed deubiquitinase with diverse effects on several cell signaling pathways (64, 65, 66). Using transiently cotransfected HEK293T cells, we demonstrated both co-IP of TRPV4^WT^ and OTUB2 and that overexpression of OTUB2 results in a reduction in TRPV4^WT^ ubiquitination levels. To our knowledge, these findings represent the first identification of a deubiquitinating interactor of TRPV4 ion channels. Together, these findings underscore the importance of protein-protein interactions mediated by the TRPV4 N-terminal domain in altering TRPV4 ubiquitination status. A key focus of future studies will be to undertake further functional assessments, including single-cell electrophysiological approaches, characterizing the modulatory effects of enzymes such as NEDD4L and OTUB2 on TRPV4 channel activity.

One of the principal goals of this study was to gain insights into potential effects of neuromuscular disease-causing mutations on the TRPV4-ARD interactome. While no candidate interactors exhibiting increased binding to the two neuromuscular disease-causing ARD mutants (TRPV4^R269C^, TRPV4^R315W^) relative to the TRPV4^WT^-ARD and the skeletal dysplasia-causing mutant TRPV4^D333G^-ARD were identified, 21 proteins exhibited reduced interactions with the neuromuscular disease mutants. Of note, mutant forms of one of these proteins, the RhoGEF ARHGEF10 (67, 68, 69, 70), have previously been associated with both hereditary neuromuscular disease (47, 48, 49, 50) and acquired forms of sensory neuropathy (71, 72). Mutations in ARHGEF10 have also been associated with a form of hereditary neuromuscular disease in Leonberger and Saint Bernard dogs that includes vocal fold paresis (51), a characteristic feature of TRPV4-mediated neuromuscular disease in human patients (11, 17, 20). Our experiments in transiently transfected MN-1 cells revealed that neuromuscular disease-causing mutations perturb the capacity of TRPV4 to immunoprecipitate with ARHGEF10. In parallel, we have demonstrated that ARHGEF10 overexpression suppresses TRPV4^WT^-mediated calcium responses to an activating stimulus, and that this modulatory effect is reduced for both TRPV4^R269C^ and TRPV4^R315W^. These findings mirror those we have described previously for RhoA, which also exerts inhibitory effects on TRPV4^WT^ channels that are diminished by neuromuscular disease-causing TRPV4 mutations (12, 28). Analysis of cryo-EM structures of the TRPV4-RhoA complex have revealed that most TRPV4-ARD residues mutated in neuromuscular disease participate in RhoA binding (12). Given that ARHGEF10 and RhoA exert similar inhibitory effects on TRPV4 channel activity and also interact functionally with one another (67, 69, 70), an important focus of future research will be to understand how the interplay between these three interactors regulates both TRPV4 and RhoA activity levels, as well as how alterations in these modulatory pathways may contribute to neuromuscular disease.

## Experimental procedures

### Expression constructs and recombinant proteins

DNA fragments corresponding to the human TRPV4^WT^-ARD (amino acids 149–397) or full N terminus (IDR and ARD, amino acids 1–397) were amplified by PCR from human TRPV4 cDNA (5) and subcloned into the Ndel and NotI sites of a pET vector with a C-terminal six-histidine epitope tag (9), then further modified to include a V5 tag C-terminal of the His tag (full V5 tag coding sequence: GCGGCCGCTCATCATCACCATCATCATGGTAAACCGATTCCGAACCCGCTGCTGGGCCTGGATAGCACCTGA; amino acid sequence: AAAHHHHHHGKPIPNPLLGLDST). Neuromuscular disease- (R269C, R315W) and skeletal dysplasia- (D333G) causing mutations were introduced using the QuikChange site-directed mutagenesis kit (Agilent) and confirmed by Sanger sequencing. Recombinant proteins were expressed in *Escherichia coli* BL21(DE3) and purified as described (9) (Fig. 1*C*). The properties of purified WT and mutant human TRPV4-ARD constructs have been analyzed extensively in our previous structural and biochemical studies (5, 9, 29). Circular dichroism spectroscopy confirms that these constructs are predominantly α-helical (29), as expected for an ARD.

### Human protein microarray

Protein microarray experiments were performed utilizing the HuProt Human Proteome Microarray v2.0 (CDI Laboratories) which contains >17,000 unique full-length human proteins in duplicate (73). Binding experiments were conducted utilizing the full TRPV4^WT^ N-terminal domain (n = 2 microarray slides), or the four TRPV4-ARD constructs (WT, R269C, R315W, D333G; n = 3 microarray slides per condition). Protein microarray slides were initially incubated in blocking buffer (150 mM NaCl, 2.7 mM KCl, 25 mM Trizma base, 0.05% Tween-20, 2% bovine serum albumin, pH 7.4) for 1 h at 4 °C. Slides were then incubated with TRPV4 proteins diluted to 1 μM in assay buffer (150 mM NaCl, 2.7 mM KCl, 25 mM Trizma base, 0.05% Tween-20, pH 7.4) for 1 h at 4 °C. Unbound proteins were then removed by three 10-min washes in assay buffer, and the slides incubated for 1 h at 4 °C in Alexa Fluor 647-conjugated mouse anti-V5 monoclonal antibody (1:1000; Thermo Fisher Scientific, 451098, RRID: AB_2532221) diluted in blocking buffer. A negative control experiment was also performed in parallel in which the anti-V5 antibody was applied without prior incubation of TRPV4 proteins. Following three 10-min washes in assay buffer, and one 10-min rinse in dH_2_O, the microarray slides were air dried and scanned using a GenePix 4000B scanner (Molecular Devices) supported with GenePix Pro 7 software (www.moleculardevices.com). Initially, damaged spots or spots in high-background regions of the slide were first identified as having a coefficient of variation value > 1.5 and excluded from analysis. A background correction was then applied to the remaining spots, by dividing the foreground median signal intensity for each spot by the background median intensity. Candidate binding partners of the full TRPV4^WT^ N-terminus were identified as proteins for which the signal intensities of the duplicate spots on both microarrays were ≥2 SDs above the mean value (*i*.*e*.*, z*-score ≥2). For the four TRPV4-ARD constructs, candidate binding partners were identified as proteins for which, on at least two of the three experiments performed with each construct, the average signal intensity of the duplicate spots was ≥2 SDs above the mean value (*i*.*e*,. *z*-score ≥2). Due to the well-established localization of TRPV4 to the plasma membrane (13), candidate interactors that localize solely to nuclear, inner mitochondrial, or extracellular compartments (TRPV4^WT^ N terminus, n = 2 proteins; TRPV4^WT^-ARD, n = 26 proteins) were excluded from further analysis, but are listed in Table S1). No positive hits were detected on the negative control microarray slides. The lists of ARD candidate binding partners were used to identify significantly enriched Gene Ontology (GO) categories (Cellular Component, Molecular Function) using the PANTHER database (PANTHER v.19.0; www.pantherdb.org) (74, 75). Enrichment analyses of structural domains were performed using the STRING database (STRING v.12.0; www.string-db.org) (76) to examine UniProt “Family & Domain” Annotated Keywords. Both sets of analyses were performed utilizing a reference list comprising the proteins present on the HuProt Human Proteome Microarray v2.0^73^. A threshold of 0.01 (FDR < 0.01) was used to identify significantly enriched GO categories or UniProt Annotated Keywords. Protein-protein interactions between candidate binding partners of the TRPV4-ARD were mapped using the STRING database (STRING v.12.0; www.string-db.org) (76) utilizing a high confidence interaction score of >0.9. Identification of conserved protein sequences amongst TRPV4-ARD candidate binding partners was performed using the PRATT software tool (PRATT v2.1; https://web.expasy.org/pratt/), with potential enrichment of identified sequences assessed using the ScanProsite software tool (https://prosite.expasy.org/scanprosite/).

### Coimmunoprecipitation and immunoblotting

HEK293T cells (ATCC, #CRL-3216) and MN-1 cells (mouse motor neuron-neuroblastoma fusion cell line (52)) were cultured in Dulbecco’s Modified Eagle Medium (Thermo Fisher Scientific) supplemented with 10% (v/v) fetal bovine serum (Thermo Fisher Scientific) and 1x penicillin/streptomycin (Thermo Fisher Scientific) at 37 °C with 5% CO_2_. Cells were cotransfected using Lipofectamine LTX and PLUS Reagent (Thermo Fisher Scientific) with pcDNA3-mCherry (5), pcDNA3.1-TRPV4-FLAG, or -GFP (WT or mutant (5, 25, 28)) and the following constructs: PSD95-FLAG (Addgene, #15463, RRID:Addgene_15463), OTUB2-V5 (Thermo Fisher Scientific, Ultimate ORF IOH45933 cloned into pCAGIG-V5 (77) using Gateway cloning), HA-NEDD4L (Addgene, #27000, RRID:Addgene_27000), HA-NEDD4L-DD (Addgene, #27001, RRID:Addgene_27001), EGFP (Clontech, pEGFP-C1), or ARHGEF10-GFP and ARHGEF10-Myc (kindly provided by Shinobu Inagaki and Satoshi Shibata, University of Osaka). To limit calcium cytotoxicity, the TRPV4-specific antagonist GSK2193874 (500 nM; Millipore Sigma, SML0942) or HC-067047 (500 nM; Millipore Sigma, 616521) was added at 4 h post-transfection. At 24 h post-transfection, cells were lysed on ice for 15 min in Pierce IP Lysis Buffer (Thermo Fisher Scientific, #87787) supplemented with EDTA-free Halt Protease and Phosphatase Inhibitor Cocktail (Thermo Fisher Scientific, #78440) and the lysates then centrifuged at 21,400×*g* for 15 minutes at 4 °C. Supernatants were incubated with primary antibody bound to Dynabeads Protein G (Thermo Fisher Scientific, 10004D) for 1 hour at 4 °C. Primary antibodies used for coimmunoprecipitation were monoclonal mouse anti-FLAG antibody (5 μg/ml; Millipore Sigma, F1804, RRID: AB_262044), monoclonal mouse anti-GFP (5 μg/ml; Thermo Fisher Scientific, A11120, RRID: AB_221568), monoclonal mouse anti-V5 (5 μg/ml; Thermo Fisher Scientific, R960–25, RRID: AB_2556564), monoclonal rat anti-HA (5 μg/ml; Sigma, 11867423001, RRID: AB_2687407), and monoclonal mouse anti-Myc (5 μg/ml; Cell Signaling Technology, 2276, RRID: AB_331783). Following several washes in IP Wash Buffer (PBS, 0.2% Tween-20), bound proteins were eluted in Elution Buffer comprising 50% (v/v) RIPA Buffer (Thermo Fisher Scientific, #R0278), 47.5% (v/v) 2x Laemmli Sample Buffer (Bio-Rad Laboratories, 1610737) and 2.5% (v/v) β-mercaptoethanol (Millipore Sigma, M3148) for 10 minutes at 70 °C. Protein lysates were resolved on 4 to 15% TGX gels (Bio-Rad Laboratories) and transferred to PVDF membranes (Thermo Fisher Scientific). Primary antibodies used for immunoblotting were monoclonal rabbit anti-FLAG (1:1000; Cell Signaling Technology, 2368, RRID: AB_2217020), polyclonal rabbit anti-GFP (1:1000; Thermo Fisher Scientific, A11122, RRID: AB_221569), polyclonal rabbit anti-Myc (1:1000, Cell Signaling Technology, 2272, RRID: AB_10692100), monoclonal mouse anti-V5 (1:1000, Thermo Fisher Scientific, R960–25), monoclonal rabbit anti-V5 (1:1000, Cell Signaling Technology, 13202, RRID: AB_2687461), and monoclonal rabbit anti-HA (1:1000, Cell Signaling Technology, 3724, AB_1549585). Secondary antibodies used were HRP-conjugated monoclonal mouse anti-rabbit IgG, light chain specific (1:150,000; Jackson ImmunoResearch, 211–032–171, RRID: AB_2339149), HRP-conjugated polyclonal goat anti-mouse IgG, light chain specific (1:150,000; Jackson ImmunoResearch, 115–035–174, RRID: AB_2338512), and HRP-conjugated polyclonal goat anti-rat IgG, light chain specific (1:150,000; Jackson ImmunoResearch, 112–035–175, RRID: AB_2338140). Membranes were developed using SuperSignal West Femto Maximum Sensitivity Substrate (Thermo Fisher Scientific) and imaged using an ImageQuant LAS 4000 system (GE Healthcare).

### Ubiquitination assay

TRPV4 ubiquitination was assessed as previously described (30). Briefly, HEK293T cells were transfected as described above and then lysed at 24 h post-transfection in RIPA Buffer supplemented with EDTA-free Halt Protease and Phosphatase Inhibitor Cocktail. For cells overexpressing NEDD4L, the deubiquitinase inhibitor PR-619 (50 μM; MedChemExpress) was also included in the lysis buffer. Lysates were sonicated for 3 min in a chilled bath sonicator and then centrifuged at 21,400×*g* for 15 minutes at 4 °C. Supernatants were incubated with Dynabeads Protein G bound to monoclonal mouse anti-FLAG antibody (5 μg/ml; Millipore Sigma, F1804, RRID: AB_262044) for 1 hour at 4 °C. Following several washes in IP Wash Buffer, bound proteins were eluted as described above. Protein lysates were resolved on 4 to 15% TGX gels and transferred to PVDF membranes. The primary antibody used was a monoclonal HRP-conjugated mouse anti-Ubiquitin antibody (1:1000; Enzo Life Sciences; BML-PW0935–0025, RRID: AB_11181761). The secondary antibody used was a HRP-conjugated mouse anti-rabbit light chain-specific secondary antibody (1:150,000; Jackson ImmunoResearch, 211–032–171, RRID: AB_2339149). Membranes were developed and imaged as outlined above. TRPV4 ubiquitination levels were determined by dividing the densitometric values for the ubiquitin bands by the values of the corresponding immunoprecipitated TRPV4 bands.

### Ratiometric calcium imaging

MN-1 cells were transfected with GFP-tagged TRPV4 (WT or mutant) or EGFP (for negative control experiments), with or without Myc-tagged ARHGEF10 using Lipofectamine LTX and PLUS Reagent. Calcium imaging was performed using a Zeiss AxioObserver 7 inverted microscope as previously described (25). Briefly, cells were bath-loaded with the calcium indicator Fura-2 AM (8 μM; Thermo Fisher Scientific) for 40 min at 37 °C in Calcium Imaging Buffer (CIB: 140 mM NaCl, 5 mM KCl, 1 mM MgCl_2_, 2 mM CaCl_2_, 24 mM glucose, 10 mM HEPES, pH 7.4). For hypotonic saline treatment, one volume of NaCl-free CIB was added to one volume of standard CIB for a final NaCl concentration of 70 mM. Cells were imaged every 10 s for 20 s prior to stimulation with hypotonic saline and then imaged every 10 s for an additional 2 min. Analysis of calcium levels over time was performed as previously described (25).

### Experimental design and statistical rationale

The experimental design employed for the human protein microarray studies is represented schematically in Figure 1*D*. Selected candidate interactors were validated by coimmunoprecipitation assays, ubiquitination assays, and ratiometric calcium imaging. Densitometric analyses of immunoblot images were performed using ImageJ (NIH; https://imagej.net/ij/). Statistical tests, significance levels, and sample sizes are detailed in each table or figure legend. Statistical analyses were performed using GraphPad Prism 10.4.0 software (www.graphpad.com). Data are presented as means ± SD, with individual data points also plotted in column graphs.

## Data availability

All data are available in the main text or the supporting materials. Source data are available from the corresponding authors upon request.

## Supporting information

This article contains supporting information.

## Conflict of interest

The authors declare that they have no conflicts of interest with the contents of this article.
