# Supplementary material for: Profiling the TRPV4 ankyrin repeat domain interactome and its disruption by neuromuscular disease-causing mutations
Source: J Biol Chem. 2025 Dec 1;302(1):110991. doi: 10.1016/j.jbc.2025.110991 (PMC12796746; doi:10.1016/j.jbc.2025.110991)
Supplement: Supplementary Material 1 [file mmc1.docx]

**PROFILING THE TRPV4 ANKYRIN REPEAT DOMAIN INTERACTOME AND ITS DISRUPTION BY NEUROMUSCULAR DISEASE-CAUSING MUTATIONS**

Alexis K. Loder, Gage P. Kosmanopoulos, William H. Aisenberg, Eric Cox, Alexis R. Meeker, Seth Blackshaw, Rachelle Gaudet, Ute A. Hellmich, Brett A. McCray, Charlotte J. Sumner, and Jeremy M. Sullivan

Corresponding authors: jsulli45@jhmi.edu & csumner1@jhmi.edu

**List of included materials:**

Fig. S1

Tables S1 to S7

**
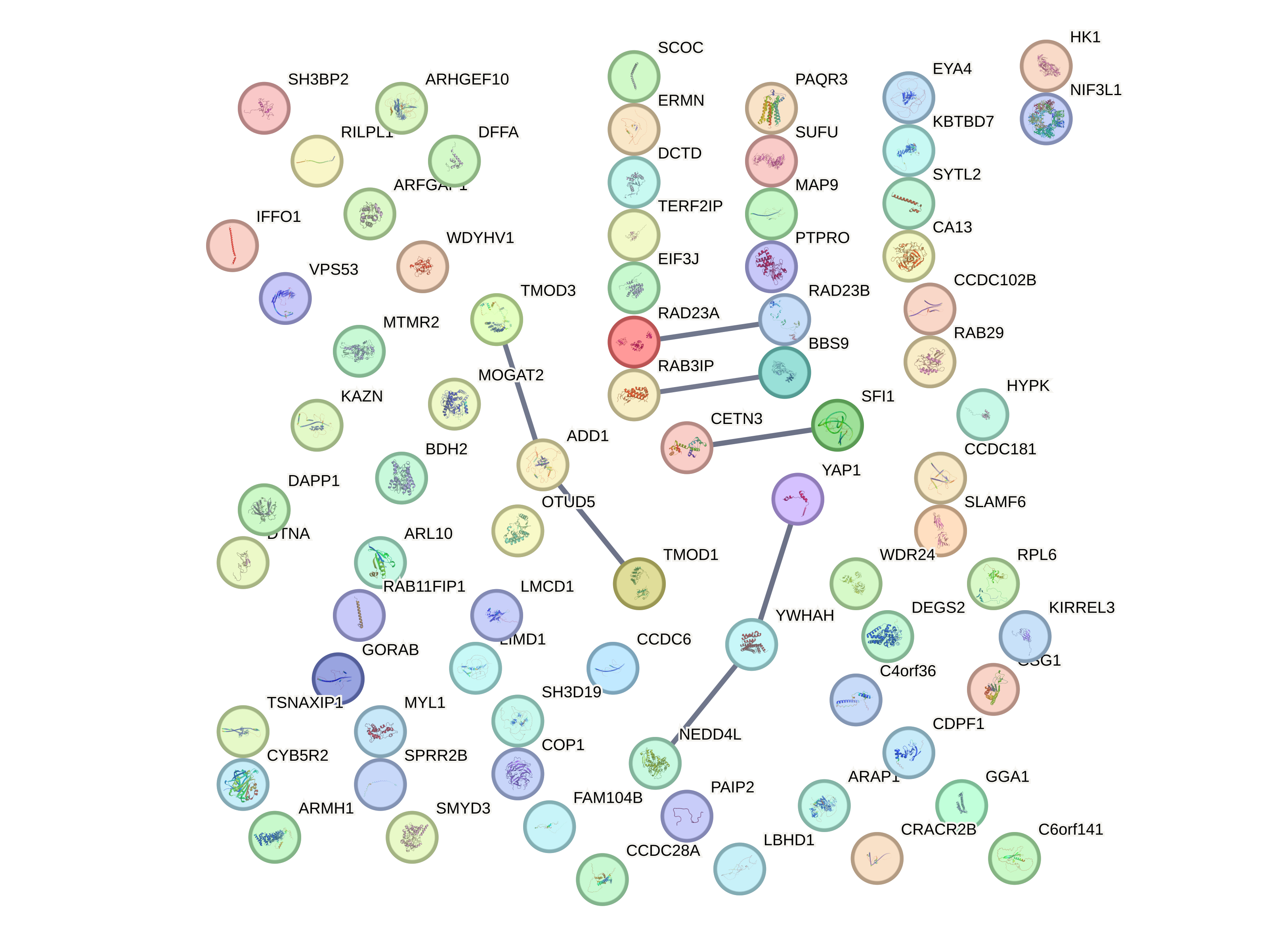
**

**Figure S1. Protein-protein interactions between microarray interactors of the TRPV4^WT^-ARD.** Interactions were visualized utilizing the STRING database (v12.0), with a minimum required interaction score of ≥ 0.9.

**Table S1.** Candidate binding partners of the TRPV4^WT^ full N-terminus and TRPV4^WT^-ARD excluded from further analysis due to their localization to the nuclear, inner mitochondrial or extracellular compartments.

| **TRPV4^WT^ full N-terminus** | | | | |
| --- | --- | --- | --- | --- |
| **Protein name**  **(UniProtKB)** | **Gene name** | **Mean *z*-score** | **SD** | **Protein localization** |
| Phosphorylated adapter RNA export protein (Q9H814) | *PHAX* | 7.82 | 2.63 | nucleus |
| Sulfite oxidase, mitochondrial (P51687) | *SUOX* | 6.33 | 5.00 | mitochondrion |
| **TRPV4^WT^-ARD** | | | | |
| **Protein name**  **(UniProtKB)** | **Gene name** | **Mean *z*-score** | **SD** | **Protein localization** |
| **RNA-binding protein 39 (**Q14498) | *RBM39* | 4.88 | 1.69 | nucleus |
| Multiple coagulation factor deficiency protein 2 (Q8NI22) | *MCFD2* | 4.45 | 0.55 | luminal (ER & Golgi) |
| U8 snoRNA-decapping enzyme (Q96DE0) | *NUDT16* | 4.42 | 1.17 | nucleus |
| Transcription regulator protein BACH1 (O14867) | *BACH1* | 3.86 | 1.83 | nucleus |
| PEST proteolytic signal-containing nuclear protein (Q8WW12) | *PCNP* | 3.71 | 0.54 | nucleus |
| **Zinc finger protein 277 (**Q9NRM2**)** | *ZNF277* | 3.51 | 0.40 | nucleus |
| **Sulfite oxidase, mitochondrial (**P51687**)** | *SUOX* | 3.49 | 0.99 | mitochondrion |
| **Zinc finger protein 225 (**Q9UK10**)** | *ZNF225* | 3.47 | 1.16 | nucleus |
| **Splicing factor 3B subunit 4 (**Q15427**)** | *SF3B4* | 3.39 | 0.59 | nucleus |
| Spermatogenesis-associated protein 20 (Q8TB22) | *SPATA20* | 3.37 | 0.89 | secreted |
| **RNA-binding protein 5 (**P52756**)** | *RBM5* | 3.35 | 0.81 | nucleus |
| Mediator of RNA polymerase II transcription subunit 8 (Q96G25) | *MED8* | 3.28 | 1.07 | nucleus |
| **Glycoprotein hormones alpha chain (**P01215**)** | *CGA* | 3.26 | 1.45 | secreted |
| Peptidyl-prolyl cis-trans isomerase E (Q9UNP9) | *PPIE* | 3.17 | 1.51 | nucleus |
| **RNA-binding protein 12 (**Q9NTZ6**)** | *RBM12* | 3.06 | 0.58 | nucleus |
| **Transcription cofactor vestigial-like protein 4** (Q14135) | *VGLL4* | 2.76 | 0.27 | nucleus |
| **Highly divergent homeobox (**Q7Z353**)** | *HDX* | 2.67 | 0.33 | nucleus |
| **T-box transcription factor TBX20 (**Q9UMR3**)** | *TBX20* | 2.64 | 0.18 | nucleus |
| Gastrin (P01350) | *GAST* | 2.55 | 0.16 | secreted |
| **Zinc finger protein 185 (**O15231**)** | *ZNF185* | 2.53 | 0.43 | nucleus |

| **Protein phosphatase 1M (**Q96MI6**)** | *PPM1M* | 2.45 | 0.04 | nucleus |
| --- | --- | --- | --- | --- |
| **Complement C2 (**P06681**)** | *C2* | 2.44 | 0.32 | secreted |
| Musculoskeletal embryonic nuclear protein 1 (Q8IVN3) | *MUSTN1* | 2.44 | 0.17 | nucleus |
| **Secretoglobin family 2A member 2 (**Q13296**)** | *SCGB2A2* | 2.36 | 0.02 | secreted |
| **RNA-binding protein 42 (**Q9BTD8**)** | *RBM42* | 2.33 | 0.18 | nucleus |
| Immunoglobulin heavy constant gamma 1 (P01857) | *IGHG1* | 2.30 | 0.21 | secreted |

SD, standard deviation.

**Table S2.** Subcellular localizations of candidate binding partners of the TRPV4^WT^-ARD*.

| **Protein (UniProtKB)** | **Gene** | **Subcellular localization** |
| --- | --- | --- |
| UV excision repair protein RAD23 homolog A (P54725) | *RAD23A* | Cytoplasm, Golgi apparatus, Proteasome |
| **SLAM family member 6** (Q96DU3) | *SLAMF6* | Plasma membrane |
| Tropomodulin-1 (P28289) | *TMOD1* | Cytoplasm, Cytoskeleton |
| Tropomodulin-3 (Q9NYL9) | *TMOD3* | Cytoplasm, Cytoskeleton |
| **Protein SFI1 homolog** (A8K8P3) | *SFI1* | Cytoplasm, Cytoskeleton |
| ADP-ribosylation factor-binding protein GGA1 (Q9UJY5) | *GGA1* | Cytoplasm, Early endosome, Golgi apparatus |
| **Protein PTHB1** (Q3SYG4) | *BBS9* | Cilium, Cytoplasm |
| Coiled-coil domain-containing protein 6 (Q16204) | *CCDC6* | Cytoplasm, Cytoskeleton |
| RAB6-interacting golgin (Q5T7V8) | *GORAB* | Cytoplasm, Golgi apparatus |
| **Transcriptional coactivator YAP1** (P46937) | *YAP1* | Cytoplasm, Plasma membrane |
| **SH3 domain-binding protein 2** (P78314) | *SH3BP2* | Unknown |
| **Suppressor of fused homolog** (Q9UMX1) | *SUFU* | Cilium, Cytoplasm |
| **Centrin-3** (O15182) | *CETN3* | Cytoplasm, Cytoskeleton |
| **Intermediate filament family orphan 1** (Q0D2I5) | *IFFO1* | Cytoplasm, Cytoskeleton |
| **Germ cell-specific gene 1 protein** (Q2KHT4) | *GSG1* | Endoplasmic reticulum |
| **Coiled-coil domain-containing protein 102B** (Q68D86) | *CCDC102B* | Cytoplasm, Cytoskeleton |
| **Hexokinase-1** (P19367) | *HK1* | Cytoplasm, Mitochondrion outer membrane |
| Protein N-terminal glutamine amidohydrolase (Q96HA8) | *WDYHV1* | Cytoplasm |
| **EF-hand calcium-binding domain-containing protein 4A** (Q8N4Y2) | *CRACR2B* | Unknown |
| **Progestin and adipoQ receptor family member 3** (Q6TCH7) | *PAQR3* | Golgi apparatus |
| Ermin (Q8TAM6) | *ERMN* | Cytoplasm, Cytoskeleton |
| Coiled-coil domain-containing protein 181 (Q5TID7) | *CCDC181* | Cytoplasm, Cytoskeleton |
| **Ras-related protein Rab-7L1** (O14966) | *RAB29* | Cytoplasm, Cytoskeleton, Endosome, Golgi apparatus |
| **Rab-3A-interacting protein** (Q96QF0) | *RAB3IP* | Cytoplasm, Cytoskeleton, Ciliary basal body, Golgi apparatus |
| **Alpha-adducin** (P35611) | *ADD1* | Cytoplasm, Cytoskeleton, Focal adhesion, Plasma membrane |
| **RILP-like protein 1** (Q5EBL4) | *RILPL1* | Cilium, Cytoplasm, Cytoskeleton, Plasma membrane |
| **OTU domain-containing protein 5** (Q96G74) | *OTUD5* | Cytoplasm |
| **Carbonic anhydrase 13** (Q8N1Q1) | *CA13* | Cytoplasm |
| **Telomeric repeat-binding factor 2-interacting protein 1** (Q9NYB0) | *TERF2IP* | Cytoplasm |
| **2-acylglycerol O-acyltransferase 2** (Q3SYC2) | *MOGAT2* | Endoplasmic reticulum |

| Dystrobrevin alpha (Q9Y4J8) | *DTNA* | Cytoplasm, Plasma membrane |
| --- | --- | --- |
| **Histone-lysine N-methyltransferase SMYD3** (Q9H7B4) | *SMYD3* | Cytoplasm |
| **Translin-associated factor X-interacting protein 1** (Q2TAA8) | *TSNAXIP1* | Cytoplasm |
| Kazrin (Q674X7) | *KAZN* | Cytoplasm, Cytoskeleton, Plasma membrane |
| **Rho guanine nucleotide exchange factor 10** (O15013) | *ARHGEF10* | Cytoplasm |
| **ADP-ribosylation factor GTPase-activating protein 1** (Q8N6T3) | *ARFGAP1* | Cytoplasm, Golgi apparatus |
| **60S ribosomal protein L6** (Q02878) | *RPL6* | Cytoplasm, Endoplasmic reticulum, Focal adhesion |
| **GATOR complex protein WDR24** (Q96S15) | *WDR24* | Cytoplasm, Lysosome |
| **DNA fragmentation factor subunit alpha** (O00273) | *DFFA* | Cytoplasm |
| Ciliary microtubule inner protein 4 (O43247) | *CIMIP4* | Flagellum |
| **Short coiled-coil protein** (Q9UIL1) | *SCOC* | Cytoplasm, Golgi apparatus |
| **Dual adapter for phosphotyrosine and 3-phosphotyrosine and 3-phosphoinositide** (Q9UN19) | *DAPP1* | Cytoplasm, Plasma membrane |
| **Uncharacterized protein C6orf141** (Q5SZD1) | *C6orf141* | Unknown |
| **Microtubule-associated protein 9** (Q49MG5) | *MAP9* | Cytoplasm, Cytoskeleton, Golgi apparatus |
| Armadillo-like helical domain containing protein 1 (Q6PIY5) | *ARMH1* | Unknown |
| **Eukaryotic translation initiation factor 3 subunit J** (O75822) | *EIF3J* | Cytoplasm |
| **Coiled-coil domain-containing protein 28A** (Q8IWP9) | *CCDC28A* | Unknown |
| **Myotubularin-related protein 2** (Q13614) | *MTMR2* | Cytoplasm, Endosome |
| **Sphingolipid delta(4)-desaturase/C4-monooxygenase DES2** (Q6QHC5) | *DEGS2* | Endoplasmic reticulum |
| **3-hydroxybutyrate dehydrogenase type 2** (Q9BUT1) | *BDH2* | Cytoplasm |
| **Synaptotagmin-like protein 2** (Q9HCH5) | *SYTL2* | Cytoplasm, Plasma membrane |
| **E3 ubiquitin-protein ligase NEDD4-like** (Q96PU5) | *NEDD4L* | Cytoplasm, Endosome, Golgi apparatus, Plasma membrane |
| **ADP-ribosylation factor-like protein 10** (Q8N8L6) | *ARL10* | Peroxisome |
| **Huntingtin-interacting protein K** (Q9NX55) | *HYPK* | Cytoplasm |
| **Arf-GAP with Rho-GAP domain, ANK repeat and PH domain-containing protein 1** (Q96P48) | *ARAP1* | Cytoplasm, Endosome, Golgi apparatus, Plasma membrane |
| **SH3 domain-containing protein 19** (Q5HYK7) | *SH3D19* | Cytoplasm, Plasma membrane |
| LOC554174 protein (Q96GL5) | LOC554174 | Unknown |
| **Deoxycytidylate deaminase** (P32321) | *DCTD* | Cytoplasm |

| **Kelch repeat and BTB domain-containing protein 7** (Q8WVZ9) | *KBTBD7* | Cytoplasm |
| --- | --- | --- |
| **LIM domain-containing protein 1** (Q9UGP4) | *LIMD1* | Cytoplasm, Focal adhesion, Plasma membrane |
| **14-3-3 protein eta** (Q04917) | *YWHAH* | Cytoplasm, Plasma membrane |
| **Protein FAM104B** (Q5XKR9) | *FAM104B* | Unknown |
| **LBH domain-containing protein 1** (Q9BQE6) | *LBHD1* | Unknown |
| **NADH-cytochrome b5 reductase 2** (Q6BCY4) | *CYB5R2* | Endoplasmic reticulum |
| **Cysteine-rich DPF motif domain-containing protein 1** (Q6NVV7) | *CDPF1* | Unknown |
| **Myosin light chain 1/3, skeletal muscle isoform** (P05976) | *MYL1* | Cytoplasm |
| **Eyes absent homolog 4** (O95677) | *EYA4* | Cytoplasm |
| **Kin of IRRE-like protein 3** (Q8IZU9) | *KIRREL3* | Plasma membrane |
| **UV excision repair protein RAD23 homolog B** (P54727) | *RAD23B* | Cytoplasm, Proteasome |
| **Uncharacterized protein C4orf36** (Q96KX1) | *C4orf36* | Unknown |
| **Small proline-rich protein 2B** (P35325) | *SPRR2B* | Cytoplasm |
| **E3 ubiquitin-protein ligase COP1** (Q8NHY2) | *COP1* | Cytoplasm |
| **NIF3-like protein 1** (Q9GZT8) | *NIF3L1* | Cytoplasm |
| **LIM and cysteine-rich domains protein 1** (Q9NZU5) | *LMCD1* | Cytoplasm |
| **Polyadenylate-binding protein-interacting protein 2** (Q9BPZ3) | *PAIP2* | Cytoplasm |
| Rab11 family-interacting protein 1 (Q6WKZ4) | *RAB11FIP1* | Cytoplasm, Endosome |
| Vacuolar protein sorting-associated protein 53 homolog (Q5VIR6) | *VPS53* | Cytoplasm, Endosome, Golgi apparatus |
| Receptor-type tyrosine-protein phosphatase O (Q16827) | *PTPRO* | Plasma membrane |

* Excluding localization to the nuclear, inner mitochondrial, or extracellular compartments.

**Table S3.** Protein domains present in candidate binding partners of the TRPV4^WT^-ARD.

| **Protein (UniProtKB)** | **Gene** | **Protein domains** |
| --- | --- | --- |
| UV excision repair protein RAD23 homolog A (P54725) | *RAD23A* | UBA, Ubiquitin-like |
| **SLAM family member 6** (Q96DU3) | *SLAMF6* | Ig-like, ITSM, Transmembrane |
| Tropomodulin-1 (P28289) | *TMOD1* | Tropomyosin-binding |
| Tropomodulin-3 (Q9NYL9) | *TMOD3* | - |
| **Protein SFI1 homolog** (A8K8P3) | *SFI1* | HAT |
| ADP-ribosylation factor-binding protein GGA1 (Q9UJY5) | *GGA1* | GAE, GAT, VHS |
| **Protein PTHB1** (Q3SYG4) | *BBS9* | - |
| Coiled-coil domain-containing protein 6 (Q16204) | *CCDC6* | Coiled coil, SH3-binding |
| RAB6-interacting golgin (Q5T7V8) | *GORAB* | Coiled coil |
| **Transcriptional coactivator YAP1** (P46937) | *YAP1* | Coiled coil, WW |
| **SH3 domain-binding protein 2** (P78314) | *SH3BP2* | SH2, SH3-binding, PH |
| **Suppressor of fused homolog** (Q9UMX1) | *SUFU* | - |
| **Centrin-3** (O15182) | *CETN3* | EF-hand |
| **Intermediate filament family orphan 1** (Q0D2I5) | *IFFO1* | Coiled coil, IF rod |
| **Germ cell-specific gene 1 protein** (Q2KHT4) | *GSG1* | Transmembrane |
| **Coiled-coil domain-containing protein 102B** (Q68D86) | *CCDC102B* | Coiled coil |
| **Hexokinase-1** (P19367) | *HK1* | Hexokinase |
| Protein N-terminal glutamine amidohydrolase (Q96HA8) | *WDYHV1* | - |
| **EF-hand calcium-binding domain-containing protein 4A** (Q8N4Y2) | *CRACR2B* | Coiled coil, EF-hand |
| **Progestin and adipoQ receptor family member 3** (Q6TCH7) | *PAQR3* | Transmembrane |
| Ermin (Q8TAM6) | *ERMN* | - |
| Coiled-coil domain-containing protein 181 (Q5TID7) | *CCDC181* | Coiled coil |
| **Ras-related protein Rab-7L1** (O14966) | *RAB29* | - |
| **Rab-3A-interacting protein** (Q96QF0) | *RAB3IP* | Coiled coil |
| **Alpha-adducin** (P35611) | *ADD1* | - |
| **RILP-like protein 1** (Q5EBL4) | *RILPL1* | Coiled coil, RH1, RH2 |
| **OTU domain-containing protein 5** (Q96G74) | *OTUD5* | OTU |
| **Carbonic anhydrase 13** (Q8N1Q1) | *CA13* | Alpha carbonic anhydrase |
| **Telomeric repeat-binding factor 2-interacting protein 1** (Q9NYB0) | *TERF2IP* | BRCT, Myb-like |
| **2-acylglycerol O-acyltransferase 2** (Q3SYC2) | *MOGAT2* | Transmembrane |
| Dystrobrevin alpha (Q9Y4J8) | *DTNA* | Coiled coil, Zinc finger |
| **Histone-lysine N-methyltransferase SMYD3** (Q9H7B4) | *SMYD3* | SET, Zinc finger |

| **Translin-associated factor X-interacting protein 1** (Q2TAA8) | *TSNAXIP1* | Coiled coil |
| --- | --- | --- |
| Kazrin (Q674X7) | *KAZN* | Coiled coil, SAM |
| **Rho guanine nucleotide exchange factor 10** (O15013) | *ARHGEF10* | Coiled coil, DH |
| **ADP-ribosylation factor GTPase-activating protein 1** (Q8N6T3) | *ARFGAP1* | Arf-GAP, Zinc finger |
| **60S ribosomal protein L6** (Q02878) | *RPL6* | - |
| **GATOR complex protein WDR24** (Q96S15) | *WDR24* | WD repeat, Zinc finger |
| **DNA fragmentation factor subunit alpha** (O00273) | *DFFA* | CIDE-N |
| Ciliary microtubule inner protein 4 (O43247) | *CIMIP4* | - |
| **Short coiled-coil protein** (Q9UIL1) | *SCOC* | Coiled coil |
| **Dual adapter for phosphotyrosine and 3-phosphotyrosine and 3-phosphoinositide** (Q9UN19) | *DAPP1* | PH, SH2 |
| **Uncharacterized protein C6orf141** (Q5SZD1) | *C6orf141* | - |
| **Microtubule-associated protein 9** (Q49MG5) | *MAP9* | Coiled coil |
| Armadillo-like helical domain containing protein 1 (Q6PIY5) | *ARMH1* | - |
| **Eukaryotic translation initiation factor 3 subunit J** (O75822) | *EIF3J* | Coiled coil |
| **Coiled-coil domain-containing protein 28A** (Q8IWP9) | *CCDC28A* | Coiled coil |
| **Myotubularin-related protein 2** (Q13614) | *MTMR2* | Coiled coil, GRAM, Myotubularin phosphatase |
| **Sphingolipid delta(4)-desaturase/C4-monooxygenase DES2** (Q6QHC5) | *DEGS2* | Transmembrane |
| **3-hydroxybutyrate dehydrogenase type 2** (Q9BUT1) | *BDH2* | - |
| **Synaptotagmin-like protein 2** (Q9HCH5) | *SYTL2* | C2, RabBD |
| **E3 ubiquitin-protein ligase NEDD4-like** (Q96PU5) | *NEDD4L* | C2, HECT, WW |
| **ADP-ribosylation factor-like protein 10** (Q8N8L6) | *ARL10* | - |
| **Huntingtin-interacting protein K** (Q9NX55) | *HYPK* | Coiled coil |
| **Arf-GAP with Rho-GAP domain, ANK repeat and PH domain-containing protein 1** (Q96P48) | *ARAP1* | Arf-GAP, PH, Ras-associating, Rho-GAP, SAM, Zinc finger |
| **SH3 domain-containing protein 19** (Q5HYK7) | *SH3D19* | SH3 |
| LOC554174 protein (Q96GL5) | LOC554174 | - |
| **Deoxycytidylate deaminase** (P32321) | *DCTD* | CMP/dCMP-type deaminase |
| **Kelch repeat and BTB domain-containing protein 7** (Q8WVZ9) | *KBTBD7* | BTB, Kelch |

| **LIM domain-containing protein 1** (Q9UGP4) | *LIMD1* | Zinc finger (LIM domain) |
| --- | --- | --- |
| **14-3-3 protein eta** (Q04917) | *YWHAH* | - |
| **Protein FAM104B** (Q5XKR9) | *FAM104B* | - |
| **LBH domain-containing protein 1** (Q9BQE6) | *LBHD1* | LBH |
| **NADH-cytochrome b5 reductase 2** (Q6BCY4) | *CYB5R2* | FAD-binding |
| **Cysteine-rich DPF motif domain-containing protein 1** (Q6NVV7) | *CDPF1* | - |
| **Myosin light chain 1/3, skeletal muscle isoform** (P05976) | *MYL1* | EF-hand |
| **Eyes absent homolog 4** (O95677) | *EYA4* | - |
| **Kin of IRRE-like protein 3** (Q8IZU9) | *KIRREL3* | Ig-like, Transmembrane |
| **UV excision repair protein RAD23 homolog B** (P54727) | *RAD23B* | STI1, UBA, Ubiquitin-like |
| **Uncharacterized protein C4orf36** (Q96KX1) | *C4orf36* | - |
| **Small proline-rich protein 2B** (P35325) | *SPRR2B* | - |
| **E3 ubiquitin-protein ligase COP1** (Q8NHY2) | *COP1* | Coiled coil, WD repeat, Zinc finger |
| **NIF3-like protein 1** (Q9GZT8) | *NIF3L1* | - |
| **LIM and cysteine-rich domains protein 1** (Q9NZU5) | *LMCD1* | LIM zinc-binding |
| **Polyadenylate-binding protein-interacting protein 2** (Q9BPZ3) | *PAIP2* | PAM |
| Rab11 family-interacting protein 1 (Q6WKZ4) | *RAB11FIP1* | C2, FIP-RBD |
| Vacuolar protein sorting-associated protein 53 homolog (Q5VIR6) | *VPS53* | Coiled coil |
| Receptor-type tyrosine-protein phosphatase O (Q16827) | *PTPRO* | Fibronectin type III, Transmembrane, Tyrosine-protein phosphatase |

Abbreviations:

Arf, ADP ribosylation factor

BRCT, BRCA1 C-terminal

CIDE, Cell death-inducing DNA fragmentation factor alpha-like effector

dCMP, Deoxycytidine monophosphate

DH, Dbl homology

FAD, flavin adenine dinucleotide

FIP, Family of interacting proteins

GAE, Gamma-adaptin ear

GAP, GTPase‐activating protein

GAT, GGA and Tom1

GRAM, Glucosyltransferases, Rab-like GTPase activators and Myotubularins

HAT, Histone acetyltransferase

HECT, Homologous to the E6-AP Carboxyl Terminus

IF, Intermediate filament

Ig, Immunoglobulin

ITSM, Immunoreceptor tyrosine-based switch motif

LBH, Limb-bud and heart

LIM, LIN-11, Isl-1 and MEC-3

OTU, Ovarian tumor

PAM, PCI/PINT-associated module

PH, Pleckstrin homology

RabBD, Rab-binding domain

RBD, Rab11-binding domain

RH1, RILP Homology 1

RH2, RILP Homology 2

SAM, Sterile alpha motif

SH2, Src Homology 2

SH3, Src Homology 3

STI1, Stress Inducible 1

UBA, Ubiquitin-associated

VHS, VPS27, Hrs and STAM

**Table S4.** Molecular function of candidate binding partners of the TRPV4^WT^-ARD.

| **Protein (UniProtKB)** | **Gene** | **Molecular function** |
| --- | --- | --- |
| UV excision repair protein RAD23 homolog A (P54725) | *RAD23A* | DNA binding, Ubiquitin binding |
| **SLAM family member 6** (Q96DU3) | *SLAMF6* | Receptor |
| Tropomodulin-1 (P28289) | *TMOD1* | Actin binding, Tropomysin binding |
| Tropomodulin-3 (Q9NYL9) | *TMOD3* | Actin binding, Cadherin binding, Tropomysin binding |
| **Protein SFI1 homolog** (A8K8P3) | *SFI1* | Phosphatase binding |
| ADP-ribosylation factor-binding protein GGA1 (Q9UJY5) | *GGA1* | Phosphatidylinositol binding, Small GTPase binding, Ubiquitin binding |
| **Protein PTHB1** (Q3SYG4) | *BBS9* | Unknown |
| Coiled-coil domain-containing protein 6 (Q16204) | *CCDC6* | Cell cycle regulation, SH3 domain binding |
| RAB6-interacting golgin (Q5T7V8) | *GORAB* | Unknown |
| **Transcriptional coactivator YAP1** (P46937) | *YAP1* | Proline-rich region binding, Transcriptional regulator |
| **SH3 domain-binding protein 2** (P78314) | *SH3BP2* | Phosphotyrosine residue binding, SH3 domain binding |
| **Suppressor of fused homolog** (Q9UMX1) | *SUFU* | Beta-catenin binding, Protein kinase binding, Transcriptional regulator |
| **Centrin-3** (O15182) | *CETN3* | Calcium ion binding, Microtubule binding |
| **Intermediate filament family orphan 1** (Q0D2I5) | *IFFO1* | Unknown |
| **Germ cell-specific gene 1 protein** (Q2KHT4) | *GSG1* | RNA polymerase binding |
| **Coiled-coil domain-containing protein 102B** (Q68D86) | *CCDC102B* | Protein serine/threonine kinase binding |
| **Hexokinase-1** (P19367) | *HK1* | ATP binding, hexokinase activity |
| Protein N-terminal glutamine amidohydrolase (Q96HA8) | *WDYHV1* | Protein-N-terminal asparagine amidohydrolase activity |
| **EF-hand calcium-binding domain-containing protein 4A** (Q8N4Y2) | *CRACR2B* | Calcium ion binding |
| **Progestin and adipoQ receptor family member 3** (Q6TCH7) | *PAQR3* | Membrane-membrane adaptor activity, Receptor, Ubiquitin-like ligase-substrate adaptor activity |
| Ermin (Q8TAM6) | *ERMN* | Actin binding |
| Coiled-coil domain-containing protein 181 (Q5TID7) | *CCDC181* | Microtubule binding |
| **Ras-related protein Rab-7L1** (O14966) | *RAB29* | GTPase activity, Kinesin binding |
| **Rab-3A-interacting protein** (Q96QF0) | *RAB3IP* | GTPase binding, Guanine nucleotide exchange factor (GEF) |
| **Alpha-adducin** (P35611) | *ADD1* | Actin binding, Cadherin binding, Calmodulin binding, RNA binding |
| **RILP-like protein 1** (Q5EBL4) | *RILPL1* | Small GTPase binding |
| **OTU domain-containing protein 5** (Q96G74) | *OTUD5* | Deubiquitinase activity |
| **Carbonic anhydrase 13** (Q8N1Q1) | *CA13* | Carbonate dehydratase activity, Zinc ion binding |
| **Telomeric repeat-binding factor 2-interacting protein 1** (Q9NYB0) | *TERF2IP* | DNA binding, Phosphatase binding |
| **2-acylglycerol O-acyltransferase 2** (Q3SYC2) | *MOGAT2* | 2-acylglycerol O-acyltransferase activity |

| Dystrobrevin alpha (Q9Y4J8) | *DTNA* | PDZ domain binding |
| --- | --- | --- |
| **Histone-lysine N-methyltransferase SMYD3** (Q9H7B4) | *SMYD3* | Histone-lysine N-methyltransferase activity |
| **Translin-associated factor X-interacting protein 1** (Q2TAA8) | *TSNAXIP1* | Unknown |
| Kazrin (Q674X7) | *KAZN* | Unknown |
| **Rho guanine nucleotide exchange factor 10** (O15013) | *ARHGEF10* | Kinesin binding, Guanine nucleotide exchange factor (GEF) |
| **ADP-ribosylation factor GTPase-activating protein 1** (Q8N6T3) | *ARFGAP1* | GTPase activator activity (GAP) |
| **60S ribosomal protein L6** (Q02878) | *RPL6* | Cadherin binding, DNA binding, RNA binding |
| **GATOR complex protein WDR24** (Q96S15) | *WDR24* | Ubiquitin protein ligase activity |
| **DNA fragmentation factor subunit alpha** (O00273) | *DFFA* | Deoxyribonuclease inhibitor activity, Protein folding chaperone |
| Ciliary microtubule inner protein 4 (O43247) | *CIMIP4* | Unknown |
| **Short coiled-coil protein** (Q9UIL1) | *SCOC* | Unknown |
| **Dual adapter for phosphotyrosine and 3-phosphotyrosine and 3-phosphoinositide** (Q9UN19) | *DAPP1* | Phospholipid binding |
| **Uncharacterized protein C6orf141** (Q5SZD1) | *C6orf141* | Unknown |
| **Microtubule-associated protein 9** (Q49MG5) | *MAP9* | Calmodulin binding, Microtubule binding |
| Armadillo-like helical domain containing protein 1 (Q6PIY5) | *ARMH1* | Unknown |
| **Eukaryotic translation initiation factor 3 subunit J** (O75822) | *EIF3J* | Translation initiation factor activity |
| **Coiled-coil domain-containing protein 28A** (Q8IWP9) | *CCDC28A* | Unknown |
| **Myotubularin-related protein 2** (Q13614) | *MTMR2* | Phosphatidylinositol-3-phosphatase activity, Phosphatidylinositol-3,5-bisphosphate 3-phosphatase activity |
| **Sphingolipid delta(4)-desaturase/C4-monooxygenase DES2** (Q6QHC5) | *DEGS2* | Sphingosine hydroxylase activity |
| **3-hydroxybutyrate dehydrogenase type 2** (Q9BUT1) | *BDH2* | 3-hydroxybutyrate dehydrogenase activity, NAD binding |
| **Synaptotagmin-like protein 2** (Q9HCH5) | *SYTL2* | Phosphatase binding, Phospholipid binding, Rab GTPase binding |
| **E3 ubiquitin-protein ligase NEDD4-like** (Q96PU5) | *NEDD4L* | Ion channel binding, Ubiquitin protein ligase activity |
| **ADP-ribosylation factor-like protein 10** (Q8N8L6) | *ARL10* | GTP binding |
| **Huntingtin-interacting protein K** (Q9NX55) | *HYPK* | Protein N-terminus binding, Protein folding chaperone |
| **Arf-GAP with Rho-GAP domain, ANK repeat and PH domain-containing protein 1** (Q96P48) | *ARAP1* | GTPase activator activity, Phospholipid binding, Type 1 angiotensin receptor binding |
| **SH3 domain-containing protein 19** (Q5HYK7) | *SH3D19* | Proline-rich region binding |
| LOC554174 protein (Q96GL5) | LOC554174 | Unknown |
| **Deoxycytidylate deaminase** (P32321) | *DCTD* | dCMP deaminase activity |

| **Kelch repeat and BTB domain-containing protein 7** (Q8WVZ9) | *KBTBD7* | Ubiquitin-like ligase-substrate adaptor activity |
| --- | --- | --- |
| **LIM domain-containing protein 1** (Q9UGP4) | *LIMD1* | Transcription corepressor activity |
| **14-3-3 protein eta** (Q04917) | *YWHAH* | Actin binding, Ion channel binding |
| **Protein FAM104B** (Q5XKR9) | *FAM104B* | Unknown |
| **LBH domain-containing protein 1** (Q9BQE6) | *LBHD1* | Unknown |
| **NADH-cytochrome b5 reductase 2** (Q6BCY4) | *CYB5R2* | Cytochrome-b5 reductase activity, FAD binding |
| **Cysteine-rich DPF motif domain-containing protein 1** (Q6NVV7) | *CDPF1* | Unknown |
| **Myosin light chain 1/3, skeletal muscle isoform** (P05976) | *MYL1* | Calcium ion binding |
| **Eyes absent homolog 4** (O95677) | *EYA4* | Protein tyrosine phosphatase activity |
| **Kin of IRRE-like protein 3** (Q8IZU9) | *KIRREL3* | Unknown |
| **UV excision repair protein RAD23 homolog B** (P54727) | *RAD23B* | DNA binding, Ubiquitin binding |
| **Uncharacterized protein C4orf36** (Q96KX1) | *C4orf36* | Unknown |
| **Small proline-rich protein 2B** (P35325) | *SPRR2B* | Unknown |
| **E3 ubiquitin-protein ligase COP1** (Q8NHY2) | *COP1* | Ubiquitin protein ligase activity |
| **NIF3-like protein 1** (Q9GZT8) | *NIF3L1* | Transcriptional regulator |
| **LIM and cysteine-rich domains protein 1** (Q9NZU5) | *LMCD1* | Transcriptional regulator |
| **Polyadenylate-binding protein-interacting protein 2** (Q9BPZ3) | *PAIP2* | Translation repressor activity |
| Rab11 family-interacting protein 1 (Q6WKZ4) | *RAB11FIP1* | Rab GTPase binding |
| Vacuolar protein sorting-associated protein 53 homolog (Q5VIR6) | *VPS53* | Unknown |
| Receptor-type tyrosine-protein phosphatase O (Q16827) | *PTPRO* | Hydrolase, Protein phosphatase, Receptor |

**Table S5.** Candidate binding partners of the TRPV4^R269C^-ARD construct.

| **Protein (UniProtKB)** | **Gene** | **Mean *z*-score** | **SD** | **# of chips**  **(n=3 total)** |
| --- | --- | --- | --- | --- |
| **UV excision repair protein RAD23 homolog A** (P54725) | *RAD23A* | 11.23 | 1.26 | 3 |
| **SLAM family member 6** (Q96DU3) | *SLAMF6* | 6.44 | 3.12 | 3 |
| **Protein SFI1 homolog** (A8K8P3) | *SFI1* | 4.29 | 1.26 | 3 |
| Tropomodulin-1 (P28289) | *TMOD1* | 4.05 | 1.19 | 3 |
| Tropomodulin-3 (Q9NYL9) | *TMOD3* | 3.91 | 1.02 | 3 |
| **Ras-related protein Rab-7L1** (O14966) | *RAB29* | 3.90 | 0.62 | 2 |
| **Coiled-coil domain-containing protein 6** (Q16204) | *CCDC6* | 3.45 | 0.70 | 3 |
| **Hexokinase-1** (P19367) | *HK1* | 3.34 | 0.45 | 3 |
| **Sphingolipid delta(4)-desaturase/C4-monooxygenase DES2** (Q6QHC5) | *DEGS2* | 3.17 | 1.31 | 2 |
| **LIM domain-containing protein 1** (Q9UGP4) | *LIMD1* | 3.00 | 0.77 | 2 |
| **Leucine-zipper-like transcriptional regulator 1** (Q8N653) | *LZTR1* | 2.92 | 0.08 | 2 |
| **Platelet glycoprotein Ib alpha chain** (P07359) | *GP1BA* | 2.77 | 0.38 | 2 |
| **SPARC-like protein 1** (Q14515) | *SPARCL1* | 2.76 | 0.88 | 2 |
| **Ubiquitin thioesterase OTUB2** (Q96DC9) | *OTUB2* | 2.71 | 0.16 | 3 |
| **Rab-3A-interacting protein, Rab3A-interacting protein** (Q96QF0) | *RAB3IP* | 2.69 | 0.63 | 3 |
| **Zinc finger protein 507** (Q8TCN5) | *ZNF507* | 2.69 | 0.65 | 2 |
| **Transmembrane protein 222** (Q9H0R3) | *TMEM222* | 2.65 | 0.32 | 2 |
| **Coiled-coil domain-containing protein 181** (Q5TID7) | *CCDC181* | 2.59 | 0.71 | 2 |
| **Aflatoxin B1 aldehyde reductase member 2** (O43488) | *AKR7A2* | 2.48 | 0.31 | 2 |
| **Tumor necrosis factor receptor superfamily member 6** (P25445) | *FAS* | 2.36 | 0.21 | 2 |
| **Myotubularin-related protein 2** (Q13614) | *MTMR2* | 2.34 | 0.13 | 3 |

SD, standard deviation.

**Table S6.** Candidate binding partners of the TRPV4^R315W^-ARD construct.

| **Protein (UniProtKB)** | **Gene** | **Mean *z*-score** | **SD** | **# of chips**  **(n=3 total)** |
| --- | --- | --- | --- | --- |
| UV excision repair protein RAD23 homolog A (P54725) | *RAD23A* | 7.15 | 9.12 | 3 |
| **SLAM family member 6** (Q96DU3) | *SLAMF6* | 5.31 | 7.14 | 3 |
| Tropomodulin-1 (P28289) | *TMOD1* | 7.24 | 5.63 | 3 |
| **Protein SFI1 homolog** (A8K8P3) | *SFI1* | 8.00 | 5.43 | 3 |
| Bromodomain And WD Repeat Domain Containing 1 (Q9NSI6) | *BRWD1* | 7.97 | 5.23 | 2 |
| ADP-ribosylation factor-binding protein GGA1 (Q9UJY5) | *GGA1* | 9.68 | 5.08 | 3 |
| ADP-Ribosylhydrolase Like 1 (Q8BGK2) | *ADPRHL1* | 7.48 | 5.02 | 2 |
| Tropomodulin-3 (Q9NYL9) | *TMOD3* | 5.12 | 4.78 | 3 |
| BH3 Interacting Domain Death Agonist (P55957) | *BID* | 4.48 | 2.08 | 2 |
| EEF1A Lysine Methyltransferase 1 (Q8WVE0) | *EEF1AKMT1* | 4.22 | 2.53 | 2 |
| SH2 Domain Containing 2A (Q9NP31) | *SH2D2A* | 4.21 | 0.18 | 2 |
| COPI Coat Complex Subunit Epsilon (O14579) | *COPE* | 4.17 | 2.72 | 2 |
| Coiled-coil domain-containing protein 6 (Q16204) | *CCDC6* | 4.10 | 1.03 | 3 |
| Dystrobrevin Alpha (Q9Y4J8) | *DTNA* | 4.04 | 1.20 | 2 |
| **Intermediate filament family orphan 1** (Q0D2I5) | *IFFO1* | 4.02 | 0.39 | 3 |
| WDYHV Motif Containing 1 (Q96HA8) | *WDYHV1* | 3.90 | 2.41 | 2 |
| EPS8 Like 2 (Q9H6S3) | *EPS8L2* | 3.90 | 0.45 | 2 |
| RAB29, Member RAS Oncogene Family (O14966) | *RAB29* | 3.86 | 0.74 | 3 |
| Ubiquitin Domain Containing 2 (Q8WUN7) | *UBTD2* | 3.84 | 2.13 | 2 |
| Delta 4-Desaturase, Sphingolipid 2 (Q6QHC5) | *DEGS2* | 3.80 | 0.20 | 2 |
| Neurocalcin Delta (P61601) | *NCALD* | 3.71 | 1.43 | 2 |
| G Protein-Coupled Receptor 45 (Q9Y5Y3) | *GPR45* | 3.63 | 0.02 | 2 |
| Profilin 1 (P07737) | *PFN1* | 3.53 | 0.90 | 2 |
| LIM Domains Containing 1 (Q9UGP4) | *LIMD1* | 3.51 | 1.22 | 2 |
| RAB3A Interacting Protein (Q96QF0) | *RAB3IP* | 3.46 | 0.70 | 3 |
| Synaptotagmin Like 2 (Q9HCH5) | *SYTL2* | 3.44 | 0.39 | 2 |
| SH3 Domain Binding Protein 2 (P78314) | *SH3BP2* | 3.41 | 0.99 | 2 |
| **Protein argonaute-3** (Q9H9G7) | *AGO3* | 3.41 | 1.29 | 2 |
| Poly(A) Binding Protein Interacting Protein 2 (Q9BPZ3) | *PAIP2* | 3.40 | 0.03 | 2 |
| Yes Associated Protein 1 (P46937) | *YAP1* | 3.34 | 1.00 | 3 |
| Membrane Spanning 4-Domains A3 (Q96HJ5) | *MS4A3* | 3.30 | 0.89 | 2 |
| **Protein pitchfork** (Q8TCI5) | *PIFO* | 3.30 | 0.35 | 2 |
| Testis Expressed 33 (O43247) | *TEX33* | 3.25 | 0.57 | 3 |

| NEDD4 Like E3 Ubiquitin Protein Ligase (Q96PU5) | *NEDD4L* | 3.24 | 0.51 | 2 |
| --- | --- | --- | --- | --- |
| Chromosome 6 Open Reading Frame 141 (Q5SZD1) | *C6orf141* | 3.24 | 1.48 | 2 |
| **Cysteine--tRNA ligase, cytoplasmic** (P49589) | *CARS* | 3.23 | 0.16 | 2 |
| DCMP Deaminase (P32321) | *DCTD* | 3.23 | 1.46 | 2 |
| Brain Expressed Associated With NEDD4 1 (Q3B7T3) | *BEAN1* | 3.22 | 0.30 | 2 |
| NPL4 Homolog, Ubiquitin Recognition Factor (Q8TAT6) | *NPLOC4* | 3.20 | 0.37 | 2 |
| Lin-7 Homolog B, Crumbs Cell Polarity Complex Component (Q9HAP6) | *LIN7B* | 3.20 | 0.10 | 2 |
| Hexokinase 1 (P19367) | *HK1* | 3.17 | 0.07 | 2 |
| Prefoldin Subunit 4 (Q9NQP4) | *PFDN4* | 3.14 | 0.76 | 2 |
| Olfactory Receptor Family 52 Subfamily A Member 1 (Q9UKL2) | *OR52A1* | 3.09 | 0.77 | 2 |
| Coiled-Coil Domain Containing 181 (Q5TID7) | *CCDC181* | 3.08 | 0.86 | 2 |
| Ribosomal Protein SA (P08865) | *RPSA* | 3.03 | 0.31 | 2 |
| ADP Ribosylation Factor GTPase Activating Protein 3 (Q9NP61) | *ARFGAP3* | 3.03 | 0.44 | 2 |
| Eukaryotic Translation Termination Factor 1 (P62495) | *ETF1* | 3.02 | 0.39 | 2 |
| Coiled-Coil Domain Containing 58 (Q4VC31) | *CCDC58* | 3.00 | 0.69 | 2 |
| Biogenesis Of Lysosomal Organelles Complex 1 Subunit 4 (Q9NUP1) | *BLOC1S4* | 2.97 | 0.23 | 2 |
| Adducin 1 (P35611) | *ADD1* | 2.93 | 1.02 | 2 |
| Olfactory Receptor Family 5 Subfamily D Member 16 (Q8NGK9) | *OR5D16* | 2.93 | 1.15 | 2 |
| Rab Interacting Lysosomal Protein Like 1 (Q5EBL4) | *RILPL1* | 2.90 | 0.10 | 2 |
| Selectin P Ligand (Q14242) | *SELPLG* | 2.89 | 0.73 | 2 |
| Steroid Receptor RNA Activator 1 (Q9HD15) | *SRA1* | 2.88 | 0.66 | 2 |
| Aldo-Keto Reductase Family 7 Member A2 (O43488) | *AKR7A2* | 2.88 | 0.81 | 3 |
| RAB34, Member RAS Oncogene Family (Q9BZG1) | *RAB34* | 2.87 | 0.34 | 2 |
| Endoplasmic Reticulum Protein 27 (Q96DN0) | *ERP27* | 2.85 | 0.06 | 2 |
| Lymphocyte Antigen 6 Family Member E (Q16553) | *LY6E* | 2.84 | 0.90 | 2 |
| Dopa Decarboxylase (P20711) | *DDC* | 2.83 | 0.23 | 2 |
| Transmembrane Protein 168 (Q9H0V1) | *TMEM168* | 2.83 | 1.00 | 2 |
| Reticulophagy Regulator 1 (Q9H6L5) | *FAM134B* | 2.82 | 0.91 | 2 |
| Solute Carrier Family 1 Member 2 (P43004) | *SLC1A2* | 2.80 | 0.97 | 2 |
| Unc-13 Homolog D (Q70J99) | *UNC13D* | 2.78 | 0.30 | 2 |
| Vasohibin 2 (Q86V25) | *VASH2* | 2.76 | 0.39 | 2 |
| Adenosylmethionine Decarboxylase 1 (P17707) | *AMD1* | 2.76 | 0.68 | 2 |

| Serum/Glucocorticoid Regulated Kinase Family Member 3 (Q96BR1) | *SGK3* | 2.74 | 0.30 | 2 |
| --- | --- | --- | --- | --- |
| Olfactory Receptor Family 5 Subfamily H Member 6 (Q8NGV6) | *OR5H6* | 2.71 | 0.31 | 2 |
| Myotubularin Related Protein 2 (Q13614) | *MTMR2* | 2.70 | 0.56 | 2 |
| Zygote Arrest 1 (Q86SH2) | *ZAR1* | 2.70 | 0.85 | 2 |
| SPG21 Abhydrolase Domain Containing, Maspardin (Q9NZD8) | *SPG21* | 2.69 | 0.45 | 2 |
| Prostaglandin E Receptor 2 (P43116) | *PTGER2* | 2.67 | 0.35 | 2 |
| Annexin A2 Receptor (Q3ZCQ2) | *ANXA2R* | 2.65 | 0.80 | 2 |
| Cysteine Rich DPF Motif Domain Containing 1 (Q6NVV7) | *CDPF1* | 2.65 | 0.53 | 2 |
| EEF1A Lysine Methyltransferase 3 (Q96AZ1) | *FAM119B* | 2.65 | 0.71 | 2 |
| Kazrin, Periplakin Interacting Protein (Q674X7) | *KAZN* | 2.64 | 0.22 | 2 |
| Histone H3 Associated Protein Kinase (Q8TF76) | *HASPIN* | 2.64 | 0.48 | 2 |
| DDB1 And CUL4 Associated Factor 17 (Q5H9S7) | *DCAF17* | 2.63 | 0.29 | 2 |
| ArfGAP With RhoGAP Domain, Ankyrin Repeat And PH Domain 1 (Q96P48) | *ARAP1* | 2.61 | 0.36 | 2 |
| SUFU Negative Regulator Of Hedgehog Signaling (Q9UMX1) | *SUFU* | 2.60 | 0.47 | 2 |
| Phosducin Like 2 (Q8N4E4) | *PDCL2* | 2.60 | 0.54 | 2 |
| Keratin Associated Protein 19-1 (Q8IUB9) | *KRTAP19-1* | 2.58 | 0.60 | 2 |
| AKT Serine/Threonine Kinase 3 (Q9Y243) | *AKT3* | 2.58 | 0.27 | 2 |
| CD200 Receptor 1 (Q8TD46) | *CD200R1* | 2.57 | 0.26 | 2 |
| Zinc And Ring Finger 4 (Q8WWF5) | *ZNRF4* | 2.55 | 0.16 | 2 |
| Chromosome X Open Reading Frame 51A (A0A1B0GTR3) | *CXorf51A* | 2.53 | 0.22 | 2 |
| Coiled-Coil Domain Containing 102B (Q68D86) | *CCDC102B* | 2.52 | 0.52 | 3 |
| Dihydropyrimidinase Like 4 (O14531) | *DPYSL4* | 2.48 | 0.19 | 2 |
| Aldehyde Dehydrogenase 1 Family Member A3 (P47895) | *ALDH1A3* | 2.43 | 0.26 | 3 |
| Cholinergic Receptor Nicotinic Delta Subunit (Q07001) | *CHRND* | 2.42 | 0.37 | 2 |
| Dual Specificity Phosphatase 12 (Q9UNI6) | *DUSP12* | 2.40 | 0.04 | 2 |
| Dual Adaptor Of Phosphotyrosine And 3-Phosphoinositides 1 (Q9UN19) | *DAPP1* | 2.36 | 0.19 | 3 |
| Sirtuin 2 (Q8IXJ6) | *SIRT2* | 2.28 | 0.19 | 2 |
| TruB Pseudouridine Synthase Family Member 1 (Q8WWH5) | *TRUB1* | 2.25 | 0.06 | 2 |
| Sorting Nexin 13 (Q9Y5W8) | *SNX13* | 2.16 | 0.00 | 2 |

SD, standard deviation.

**Table S7.** Candidate binding partners of the TRPV4^D333G^-ARD construct.

| **Protein (UniProtKB)** | **Gene** | **Mean *z*-score** | **SD** | **# of chips**  **(n=3 total)** |
| --- | --- | --- | --- | --- |
| E3 ubiquitin-protein ligase NEDD4-like (Q96PU5) | *NEDD4L* | 12.85 | 7.07 | 2 |
| **Sorting nexin-9** (Q9Y5X1) | *SNX9* | 12.43 | 11.69 | 2 |
| **Cytosolic 10-formyltetrahydrofolate dehydrogenase (O75891)** | *ALDH1L1* | 10.86 | 7.12 | 2 |
| **Protein FAM104B** (Q5XKR9) | *FAM104B* | 10.81 | 2.30 | 2 |
| Telomeric repeat-binding factor 2-interacting protein 1 (Q9NYB0) | *TERF2IP* | 10.60 | 9.26 | 2 |
| Tropomodulin-1 (P28289) | *TMOD1* | 10.41 | 6.98 | 3 |
| **Kanadaptin** (Q9BWU0) | *SLC4A1AP* | 9.82 | 6.26 | 2 |
| **Transcription cofactor vestigial-like protein 4 (Q14135)** | *VGLL4* | 9.40 | 7.14 | 2 |
| **Huntingtin-interacting protein K** (Q9NX55) | *HYPK* | 9.03 | 0.07 | 2 |
| **EF-hand calcium-binding domain-containing protein 4A (Q8N4Y2)** | *CRACR2B* | 8.58 | 0.58 | 2 |
| **Electron transfer flavoprotein subunit beta (P38117)** | *ETFB* | 7.96 | 8.05 | 2 |
| **Phosphatidylinositol N-acetylglucosaminyltransferase subunit P (P57054)** | *PIGP* | 7.79 | 5.62 | 2 |
| **Transcriptional coactivator YAP1** (P46937) | *YAP1* | 7.68 | 5.96 | 3 |
| **SLAM family member 6** (Q96DU3) | *SLAMF6* | 7.56 | 3.30 | 3 |
| **SH2 domain-containing protein 2A** (Q9NP31) | *SH2D2A* | 7.25 | 6.45 | 3 |
| **Rab11 family-interacting protein 1** (Q6WKZ4) | *RAB11FIP1* | 6.93 | 3.72 | 2 |
| **ADP-ribosylation factor-binding protein GGA1** (Q9UJY5) | *GGA1* | 6.83 | 1.06 | 3 |
| **Protein SFI1 homolog** (A8K8P3) | *SFI1* | 6.80 | 1.25 | 3 |
| **Neutrophil cytosol factor 1** (P14598) | *NCF1* | 6.72 | 3.73 | 2 |
| **Serine/threonine-protein kinase 24** (Q9Y6E0) | *STK24* | 6.61 | 6.16 | 2 |
| **Melanoma-associated antigen B10** (Q96LZ2) | *MAGEB10* | 6.19 | 2.23 | 2 |
| **Tubulin polymerization-promoting protein (O94811)** | *TPPP* | 6.08 | 2.39 | 2 |
| **Testis-expressed protein 33** (O43247) | *TEX33* | 6.00 | 4.54 | 3 |
| **UV excision repair protein RAD23 homolog A (P54725)** | *RAD23A* | 5.94 | 2.34 | 3 |
| **SH3 domain-containing protein 19** (Q5HYK7) | *SH3D19* | 5.91 | 0.15 | 2 |
| **Epidermal growth factor receptor kinase substrate 8-like protein 1 (Q8TE68)** | *EPS8L1* | 5.77 | 1.08 | 2 |
| **N-terminal EF-hand calcium-binding protein 1 (Q8N987)** | *NECAB1* | 5.66 | 3.59 | 2 |
| **BAG family molecular chaperone regulator 2 (95816)** | *BAG2* | 5.55 | 4.07 | 2 |

| **UBX domain-containing protein 2A** (P68543) | *UBXD4* | 5.48 | 4.55 | 2 |
| --- | --- | --- | --- | --- |
| **Rho guanine nucleotide exchange factor 10 (O15013)** | *ARHGEF10* | 5.47 | 4.23 | 3 |
| **Zinc finger protein 185** (Q62394) | *Zfp185* | 5.32 | 4.29 | 3 |
| **Nucleolin** (P19338) | *NCL* | 5.25 | 3.85 | 2 |
| **Vasodilator-stimulated phosphoprotein (P50552)** | *VASP* | 5.23 | 0.41 | 2 |
| **Coiled-coil domain-containing protein 102B** (Q68D86) | *CCDC102B* | 5.22 | 0.78 | 3 |
| **Cyclin-dependent kinase inhibitor 1B** (P46527) | *CDKN1B* | 5.19 | 4.49 | 3 |
| **Coiled-coil domain-containing protein 6** (Q16204) | *CCDC6* | 5.17 | 1.88 | 3 |
| **Tubulinyl-Tyr carboxypeptidase 1** (Q7L8A9) | *VASH1* | 5.10 | 3.72 | 2 |
| **Guanylate-binding protein 2** (P32456) | *GBP2* | 5.07 | 2.68 | 2 |
| Tropomodulin-3 (Q9NYL9) | *TMOD3* | 5.03 | 0.50 | 3 |
| **Coatomer subunit zeta-2** (Q9P299) | *COPZ2* | 4.99 | 2.65 | 2 |
| **RILP-like protein 1** (Q5EBL4) | *RILPL1* | 4.87 | 0.14 | 3 |
| **Ubiquitin-conjugating enzyme E2 C** (O00762) | *UBE2C* | 4.86 | 3.19 | 2 |
| **Nuclear protein localization protein 4 homolog** (Q8TAT6) | *NPLOC4* | 4.80 | 0.70 | 2 |
| **ADP-ribosylation factor GTPase-activating protein 1** (Q8N6T3) | *ARFGAP1* | 4.70 | 1.04 | 3 |
| **6-phosphogluconate dehydrogenase, decarboxylating** (P52209) | *PGD* | 4.59 | 2.88 | 2 |
| **2-acylglycerol O-acyltransferase 2** (Q3SYC2) | *MOGAT2* | 4.59 | 1.10 | 2 |
| **Pyrin domain-containing protein 1** (Q8WXC3) | *PYDC1* | 4.56 | 2.39 | 2 |
| **Arf-GAP with Rho-GAP domain, ANK repeat and PH domain-containing protein 1** (Q96P48) | *ARAP1* | 4.52 | 1.60 | 2 |
| **Disabled homolog 2** (P98082) | *DAB2* | 4.45 | 1.58 | 2 |
| **Melanoma-associated antigen 9** (P43362) | *MAGEA9* | 4.43 | 0.98 | 2 |
| **LBH domain-containing protein 1** (Q9BQE6) | *LBHD1* | 4.29 | 2.60 | 3 |
| **Serine/threonine-protein kinase D2** (Q9BZL6) | *PRKD2* | 4.23 | 2.79 | 2 |
| **Neurocalcin-delta** (P61601) | *NCALD* | 4.22 | 2.00 | 2 |
| **Deoxycytidylate deaminase** (P32321) | *DCTD* | 4.13 | 1.40 | 3 |
| **Calcium/calmodulin-dependent protein kinase kinase 1** (Q8N5S9) | *CAMKK1* | 4.04 | 2.36 | 2 |
| **Protein BEAN1** (Q3B7T3) | *BEAN1* | 4.01 | 0.62 | 3 |
| **Integral membrane protein GPR137** (Q96N19) | *GPR137* | 4.00 | 1.10 | 2 |
| **PRKC apoptosis WT1 regulator protein** (Q96IZ0) | *PAWR* | 3.81 | 2.13 | 2 |
| **Tyrosine-protein kinase Fyn** (P06241) | *FYN* | 3.76 | 1.93 | 2 |
| **Steroid receptor RNA activator 1** (Q9HD15) | *SRA1* | 3.75 | 0.93 | 2 |

| **Mothers against decapentaplegic homolog 7** (O15105) | *SMAD7* | 3.72 | 0.62 | 2 |
| --- | --- | --- | --- | --- |
| **Protein FAM122B** (Q7Z309) | *FAM122B* | 3.71 | 1.10 | 3 |
| **Intermediate filament family orphan 1** (Q0D2I5) | *IFFO1* | 3.71 | 1.20 | 3 |
| **Progestin and adipoQ receptor family member 3** (Q6TCH7) | *PAQR3* | 3.65 | 0.58 | 2 |
| **Myotubularin-related protein 2** (Q13614) | *MTMR2* | 3.59 | 0.08 | 2 |
| **Pleckstrin homology domain-containing family A member 4** (Q9H4M7) | *PLEKHA4* | 3.58 | 1.99 | 2 |
| **RAB6-interacting golgin** (Q5T7V8) | *GORAB* | 3.58 | 0.93 | 3 |
| **Ermin** (Q8TAM6) | *ERMN* | 3.56 | 1.54 | 3 |
| **Keratin-associated protein 19-1** (Q8IUB9) | *KRTAP19-1* | 3.54 | 0.85 | 2 |
| **UBX domain-containing protein 6** (Q9BZV1) | *UBXN6* | 3.53 | 1.29 | 2 |
| **Profilin-1** (P07737) | *PFN1* | 3.47 | 0.75 | 2 |
| **LEM domain-containing protein 1** (Q68G75) | *LEMD1* | 3.46 | 1.80 | 2 |
| **Short coiled-coil protein** (Q9UIL1) | *SCOC* | 3.42 | 0.65 | 2 |
| **Polyadenylate-binding protein-interacting protein 2** (Q9BPZ3) | *PAIP2* | 3.42 | 0.27 | 2 |
| **Kazrin** (Q674X7) | *KAZN* | 3.39 | 0.82 | 2 |
| **3-hydroxybutyrate dehydrogenase type 2** (Q9BUT1) | *BDH2* | 3.34 | 0.75 | 2 |
| **Calponin-2** (Q99439) | *CNN2* | 3.32 | 1.69 | 2 |
| **Uncharacterized protein KIAA1143** (Q96AT1) | *KIAA1143* | 3.31 | 0.36 | 2 |
| **PH and SEC7 domain-containing protein 3** (Q9NYI0) | *PSD3* | 3.24 | 0.28 | 2 |
| **Adenylate kinase 9** (Q5TCS8) | *AK9* | 3.17 | 0.97 | 2 |
| **Aflatoxin B1 aldehyde reductase member 2** (O43488) | *AKR7A2* | 3.16 | 0.63 | 2 |
| **Sodium channel subunit beta-3** (Q9NY72) | *SCN3B* | 3.11 | 0.01 | 2 |
| **Eukaryotic translation initiation factor 3 subunit J** (O75822) | *EIF3J* | 3.06 | 0.78 | 2 |
| **Olfactory receptor 10G3** (Q8NGC4) | *OR10G3* | 3.04 | 0.23 | 2 |
| **Suppressor of fused homolog** (Q9UMX1) | *SUFU* | 3.04 | 0.31 | 3 |
| **Putative uncharacterized protein FLJ45684** (Q6ZSA8) | *FLJ45684* | 2.98 | 0.65 | 2 |
| **Centrin-3** (O15182) | *CETN3* | 2.97 | 0.49 | 2 |
| **Kin of IRRE-like protein 3** (Q8IZU9) | *KIRREL3* | 2.97 | 0.31 | 2 |
| **Glyoxylate reductase/hydroxypyruvate reductase** (Q9UBQ7) | *GRHPR* | 2.96 | 0.39 | 2 |
| **Optineurin** (Q96CV9) | *OPTN* | 2.95 | 0.59 | 2 |
| **Hexokinase-1** (P19367) | *HK1* | 2.93 | 0.51 | 2 |
| **Ras-related protein Rab-34** (Q9BZG1) | *RAB34* | 2.92 | 0.54 | 3 |
| **Rab GTPase-binding effector protein 2** (Q9H5N1) | *RABEP2* | 2.90 | 1.15 | 2 |
| **Ras-related protein Rab-40C** (Q96S21) | *RAB40C* | 2.85 | 0.42 | 2 |

| **Cell surface glycoprotein CD200 receptor 1** (Q8TD46) | *CD200R1* | 2.85 | 0.36 | 2 |
| --- | --- | --- | --- | --- |
| **Aflatoxin B1 aldehyde reductase member 3** (O95154) | *AKR7A3* | 2.81 | 0.02 | 2 |
| **BRICHOS domain-containing protein 5** (Q6PL45) | *BRICD5* | 2.81 | 0.12 | 2 |
| **Tetratricopeptide repeat protein 32** (Q5I0X7) | *TTC32* | 2.81 | 0.26 | 2 |
| **Syntaxin-16** (O14662) | *STX16* | 2.76 | 0.97 | 2 |
| **Thyroid receptor-interacting protein 6** (Q15654) | *TRIP6* | 2.76 | 0.38 | 3 |
| **Excitatory amino acid transporter 2** (P43004) | *SLC1A2* | 2.76 | 0.22 | 2 |
| **Protein FAM131B** (Q86XD5) | *FAM131B* | 2.73 | 0.16 | 2 |
| **Biogenesis of lysosome-related organelles complex 1 subunit 4** (Q9NUP1) | *BLOC1S4* | 2.72 | 0.06 | 2 |
| **Protein pitchfork** (Q8TCI5) | *PIFO* | 2.72 | 0.48 | 2 |
| **Ras-related protein M-Ras** (O14807) | *MRAS* | 2.71 | 0.43 | 2 |
| **Nuclear receptor coactivator 3** (Q9Y6Q9) | *NCOA3* | 2.71 | 0.19 | 2 |
| **DNA fragmentation factor subunit alpha** (O00273) | *DFFA* | 2.67 | 0.02 | 2 |
| **Paladin** (Q9ULE6) | *PALD1* | 2.67 | 0.84 | 2 |
| **GRIP1-associated protein 1** (Q4V328) | *GRIPAP1* | 2.66 | 0.32 | 2 |
| **Sushi repeat-containing protein SRPX2** (O60687) | *SRPX2* | 2.65 | 0.53 | 2 |
| **[F-actin]-monooxygenase MICAL1** (Q8TDZ2) | *MICAL1* | 2.64 | 0.59 | 2 |
| **Oligophrenin-1** (O60890) | *OPHN1* | 2.64 | 0.37 | 2 |
| **Keratin, type II cytoskeletal 79** (Q5XKE5) | *KRT6L* | 2.64 | 0.18 | 2 |
| **ADP-ribosylation factor-like protein 10** (Q8N8L6) | *ARL10* | 2.64 | 0.13 | 2 |
| **Tetratricopeptide repeat protein 1** (Q99614) | *TTC1* | 2.62 | 0.30 | 2 |
| **SLAIN motif-containing protein 2** (Q9P270) | *SLAIN2* | 2.62 | 0.03 | 2 |
| MGC39372 protein (Q8TB02) | *MGC39372* | 2.62 | 0.02 | 2 |
| **WD repeat-containing protein 34** (Q96EX3) | *WDR34* | 2.60 | 0.15 | 2 |
| **RNA-binding E3 ubiquitin-protein ligase MEX3C, EC 2.3.2.27** (Q5U5Q3) | *MEX3C* | 2.60 | 0.06 | 2 |
| **Neuropilin-2** (O60462) | *NRP2* | 2.59 | 0.24 | 2 |
| **Actin-related protein 2/3 complex subunit 5** (O15511) | *ARPC5* | 2.56 | 0.17 | 2 |
| **Peptidyl-prolyl cis-trans isomerase A** (P62937) | *PPIA* | 2.55 | 0.46 | 2 |
| **Tyrosine aminotransferase** (P17735) | *TAT* | 2.55 | 0.40 | 2 |
| **60S ribosomal protein L6** (Q02878) | *RPL6* | 2.55 | 0.55 | 2 |
| **Uncharacterized protein C6orf141** (Q5SZD1) | *C6orf141* | 2.54 | 0.31 | 3 |
| **N-terminal EF-hand calcium-binding protein 3** (Q96P71) | *NECAB3* | 2.50 | 0.43 | 2 |

| **cTAGE family member 6** (Q86UF2) | *CTAGE6* | 2.48 | 0.40 | 2 |
| --- | --- | --- | --- | --- |
| **DNA fragmentation factor subunit beta** (O76075) | *DFFB* | 2.48 | 0.41 | 2 |
| **Eukaryotic translation initiation factor 4H** (Q15056) | *EIF4H* | 2.44 | 0.19 | 2 |
| **Cellular nucleic acid-binding protein** (P62633) | *CNBP* | 2.40 | 0.45 | 2 |
| **Intracellular hyaluronan-binding protein 4** (Q5JVS0) | *HABP4* | 2.39 | 0.14 | 2 |
| **Rab-3A-interacting protein** (Q96QF0) | *RAB3IP* | 2.38 | 0.27 | 3 |
| **Leiomodin-1** (P29536) | *LMOD1* | 2.36 | 0.25 | 2 |
| **Cell growth regulator with RING finger domain protein 1** (Q99675) | *CGRRF1* | 2.35 | 0.08 | 2 |
| **Sorting nexin-20** (Q7Z614) | *SNX20* | 2.34 | 0.19 | 2 |
| **Cytochrome c oxidase assembly protein COX19** (Q49B96) | *COX19* | 2.10 | 0.05 | 2 |

SD, standard deviation.
